# High-throughput profiling of influenza A virus hemagglutinin gene at single-nucleotide resolution

**DOI:** 10.1038/srep04942

**Published:** 2014-05-13

**Authors:** Nicholas C. Wu, Arthur P. Young, Laith Q. Al-Mawsawi, C. Anders Olson, Jun Feng, Hangfei Qi, Shu-Hwa Chen, I.-Hsuan Lu, Chung-Yen Lin, Robert G. Chin, Harding H. Luan, Nguyen Nguyen, Stanley F. Nelson, Xinmin Li, Ting-Ting Wu, Ren Sun

**Affiliations:** 1 Department of Molecular and Medical Pharmacology, David Geffen School of Medicine, University of California, Los Angeles, CA 90095, USA; 2 Molecular Biology Institute, University of California, Los Angeles, CA 90095, USA; 3 Institute of Information Science, Academia Sinica, Taipei, Taiwan; 4 Department of Human Genetics, David Geffen School of Medicine, University of California, Los Angeles, CA 90095, USA; 5 Department of Pathology and Laboratory Medicine, David Geffen School of Medicine, University of California, Los Angeles, CA 90095, USA; 6 AIDS Institute, University of California, Los Angeles, CA 90095, USA; 7 These authors contributed equally to this work.

## Abstract

Genetic research on influenza virus biology has been informed in large part by nucleotide variants present in seasonal or pandemic samples, or individual mutants generated in the laboratory, leaving a substantial part of the genome uncharacterized. Here, we have developed a single-nucleotide resolution genetic approach to interrogate the fitness effect of point mutations in 98% of the amino acid positions in the influenza A virus hemagglutinin (HA) gene. Our HA fitness map provides a reference to identify indispensable regions to aid in drug and vaccine design as targeting these regions will increase the genetic barrier for the emergence of escape mutations. This study offers a new platform for studying genome dynamics, structure-function relationships, virus-host interactions, and can further rational drug and vaccine design. Our approach can also be applied to any virus that can be genetically manipulated.

The broad field of systems biology was significantly advanced in the past decade due to many technological improvements, such as the invention of DNA microarray, next generation sequencing, mass-spectrometry and other applications permitting high-throughput screenings^1^,^2^. These technical advancements have enabled large scale studies including interactomics, proteomics, transcriptomics, genomics, epigenomics, and metagenomics, which have revolutionized biomedical research^3^,^4^,^5^,^6^,^7^,^8^. A multitude of structure-function information is embedded in these studies that is valuable for rational drug and vaccine design. In addition, the continued development of *in silico* approaches to protein structural modeling, prediction, and design further complements the impact of high-throughput biological data^9^,^10^,^11^,^12^.

High-throughput tools have also influenced the advancement of genetic approaches. Traditional genetic methods focus on a single genotype-phenotype relationship at a time, and has been extensively employed to analyze individual mutations. In contrast, high-throughput genetic methods examine the phenotypic outcomes of multiple mutations simultaneously. Genome-wide insertional mutagenesis is a common high-throughput genetic approach. It has been employed to characterize bacterial genomes at a single-gene resolution level^13^,^14^. A higher resolution has been achieved in two medically important RNA viruses, HCV and influenza^15^,^16^. However, the maximum resolution of the insertional mutagenic approach is limited to a protein subdomain level and thus is insufficient to identify critical amino acid residues. Therefore, there is a demand for a high-throughput genetic platform at a single-residue resolution.

In this study, we developed a single-nucleotide resolution genetic approach using a large mutant library and a sensitive deep sequencing technique to annotate the influenza A virus hemagglutinin (HA) gene, which carries critical roles in receptor binding, viral entry, host shifts, and immune escape mechanisms. Here, we probe for fitness effects of individual substitutions in 98% of all amino acid positions across HA. Our results provide a comprehensive structure-function description of HA and offer a reference to identify potential vaccine epitope. More importantly, the high-throughput profiling platform established in this study can be applied to any genetically manipulable viral gene or genome to probe mutational fitness effects under any specified growth condition.

## Results

### High-throughput genetic approach at single-nucleotide resolution

**Figure 1:**
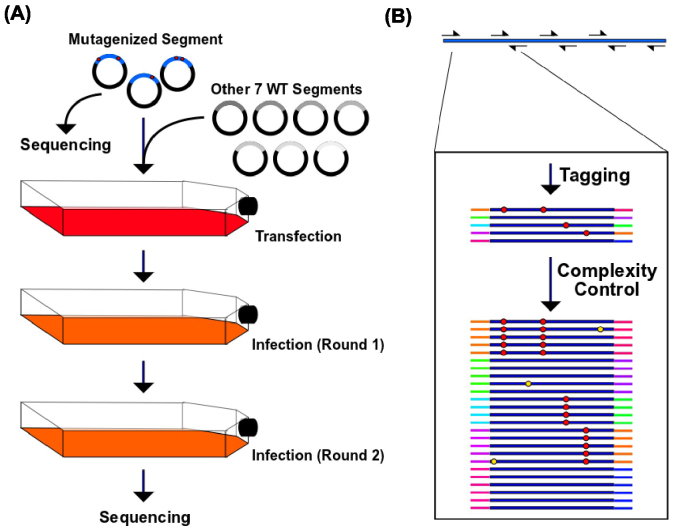
Mutant library passaging and sequencing library preparation. (A) The HA segment was randomized by error-prone PCR. The randomized segment with the remaining seven wild type segments were transfected into C227 cells to generate the viral mutant library. Two rounds of 24-hour infections were performed using A549 cells with an MOI of 0.05. Both the plasmid library and the passaged viral library were subjected to sequencing using the Illumina HiSeq 2000 machine. (B) The HA gene was divided into 12 amplicons for the first PCR. Unique tags were assigned to both ends of the individual molecules during the amplification process. The second PCR generated identical copies of individual molecules linked with unique tags. Red circles represent true mutations; Yellow circles represent sequencing errors.

The conceptual basis of our high-throughput genetic platform is to randomly mutagenize each position of the genome, monitor the enrichment or diminishment of each point mutation under a specified growth condition, and perform massive deep-sequencing to determine which mutations are associated with negative, neutral, or positive fitness outcomes under the given growth condition. The mutant library was created on influenza A/WSN/1933 (H1N1) hemagglutinin (HA) gene by performing error-prone PCR on the eight-plasmid reverse genetics system^17^ (see materials and methods). Subsequently, the viral mutant library was generated by transfection and passaged for two 24-hour replication selection rounds in A549 cells (human lung epithelial carcinoma cells) (Fig. 1A). The plasmid library and the passaged viral library were each sequenced by Illumina HiSeq 2000. Individual mutants would experience an identical selection pressure with other mutants in the pool during the course of transfection and infection. Therefore, comparing the genetic compositions of the plasmid library and the passaged viral library reflects the variation in replication rates for each mutation. Here, we use a relative fitness index (RF index) as a proxy for the fitness effect of individual mutations. The RF index is calculated as: 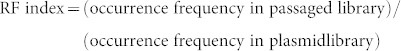
The occurrence frequency of individual mutations was largely expected to be lower than the sequencing error rate of 0.1% in the Illumina next generation sequencing (NGS).

Therefore, we utilized a two-step PCR approach for library preparation to distinguish true mutations from sequencing errors (Fig. 1B). In the first PCR, the HA gene was divided into 12 amplicons for amplification with a unique tag assigned to individual molecules. In the second PCR, multiple identical copies for individual tagged molecules were generated. The input copy number for the second PCR was well-controlled such that after a sub-saturation PCR, individual tagged molecules would be sequenced ~10 times. True mutations would exist in most, if not all, sequencing reads sharing the same tag, whereas sequencing errors would not. This error-correction approach is based on a valid assumption that occurrence of sequencing error is independent of the identity of the nucleotide tag^18^. Therefore, sequencing errors could be distinguished from true mutations. Individual molecules, each carrying a unique tag, have an average copy number of ~10 (median = 10) in the sequencing data, which verified the sequencing library preparation design.

### Point mutation fitness profiling of hemagglutinin

**Figure 2:**
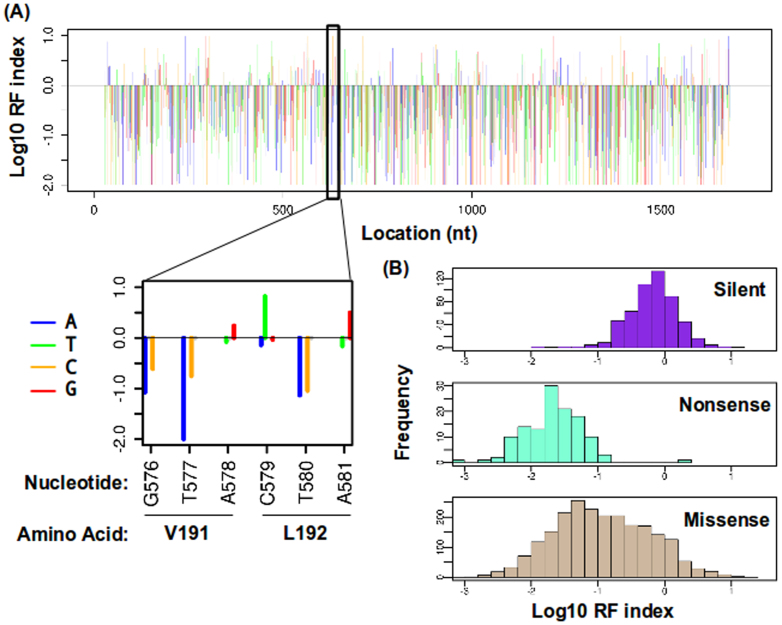
Single-nucleotide resolution fitness profiling. (A) The RF index for individual point mutations across the HA gene was computed. Log_10_ of the RF index is plotted on the y-axis. Each nucleotide position is represented by four consecutive lines for the RF indices that correspond to mutating to A (blue), T (green), C (orange), or G (red). The Log_10_ RF index of wild type (WT) nucleotides is set as zero. Only point mutations with a coverage of ≥ 30 tag-conflated reads in the plasmid library are shown. Otherwise, point mutations are plotted as a gray circle on the zero baseline. A short region is shown as an inset to demonstrate the resolution of our dataset. (B) The distributions of the log_10_ RF indices for silent substitutions, nonsense substitutions and missense substitutions are displayed as histograms. Mutations located at the 5′ terminal 200 bp and 3′ terminal 200 bp regions are not included in this analysis to avoid confounding by the vRNA packaging signal^50^.

The RF indices of individual point mutations were profiled across 98% of amino acid positions of HA in biological duplicate (Spearman correlation = 0.78) (Fig. 2A). The remaining 2% of amino acid positions not observed were from the termini of HA, where the first and last amplicon primers are located. Silent mutations and nonsense mutations provided an internal control to access the data quality. In principle, silent mutations, which alter the nucleotide sequence but not the amino acid sequence, rarely impose a fitness cost. On the other hand, nonsense mutations, which result in a truncated protein product, are lethal to the virus. Indeed, our data is consistent with this notion. Silent mutations have a significantly higher RF index than nonsense mutations (P < 2 e^−16^, two-tailed Student's t-test) (Fig. 2B). In addition, the RF index distributions of silent mutations and nonsense mutations are well separated, which validated the reliability of our approach. However, several silent mutations with a low RF index were observed, which may be indicative of their roles in codon usage, RNA structure, and other functions beyond protein-coding.

Furthermore, the fitness data is consistent with the reported phenotypes of mutants that have been previously characterized in the literature. Examples include a temperature sensitive substitution (Y174H)^19^, a host switching substitution (D238G)^20^, two ther-modynamic stabilizing substitutions (D111E and Q299R)^21^, and four HA cleavage site substitutions (Y342H, Y342C, Y342N and Y342F)^22^ (Table 1). Y174H, D238G, Y342H, Y342C, and Y342N, which are expected to be deleterious under our experimental condition (see footnote in Table 1), have a relatively low RF index (ranging from 0.04 to 0.23). On the other hand, D111E, Q299R, and Y342F, which are expected to be neutral under our experimental condition, have a relatively high RF index (ranging from 0.37 to 1.03). These comparisons show the consistency between our dataset and the experimental results reported in the literature.

**Figure 3:**
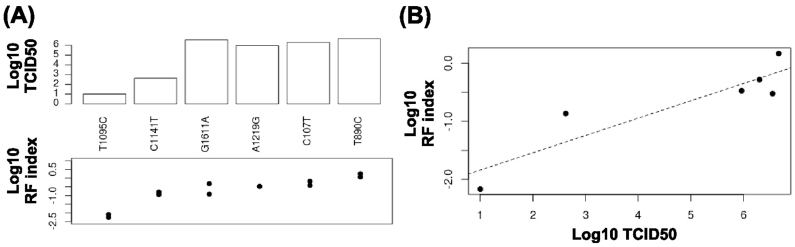
Experimental validation. (A) The top panel displays the log_10_ TCID_50_ value of mutant virus rescued from transfection. The bottom panel represents their log_10_ RF indices from the biological duplicate. (B) A Pearson correlation of 0.9 is obtained between log_10_ TCID_50_ from transfection (x-axis) and log_10_ RF index (y-axis).

Independent experimental validation also confirmed our dataset. Six randomly selected point mutations were individually reconstructed and analyzed. RF indices of each mutation have a positive correlation with the TCID_50_ value measured from a rescue experiment (Fig. 3A–B). Overall, these analyses verified the reliability of the fitness profiling data and demonstrated our platform to be comprehensive and at high resolution. The RF indices of all profiled HA amino acid substitutions are presented in Table S1.

### Structural analysis of hemagglutinin

Our platform has a high sensitivity for monitoring negative selection in addition to positive selection and therefore enables the identification of deleterious mutations that disappear throughout viral passaging. The availability of the influenza HA crystal structure allowed us to further extrapolate structural insights from our dataset. A weak, yet significant spearman correlation of 0.30 was observed between the RF index and the relative solvent accessible surface area (SASA) of HA (P < 2 e^−16^). This indicates that surface residues are more tolerant to substitutions than core residues, which is consistent with observations in cellular proteins^23^,^24^. We also analyzed the fitness effects of mutations in different types of structural elements, namely *α*-helices (mean log_10_ RF index = −1.19), *β*-strands (mean log_10_ RF index = −0.97), turns (mean log_10_ RF index = −0.98) and coils (mean log_10_ RF index = −1.01). Interestingly, mutations in *α*-helices are more deleterious than mutations in *β*-strands (P = 1 e^−4^), turns (P = 1 e^−3^) and coils (P = 2 e^−3^). In contrast, the fitness effects of mutations in *β*-strands, turns and coils are not significantly different from each other (P > 0.4). This result implies that most functional elements in HA are contained within *α*-helices.

**Figure 4:**
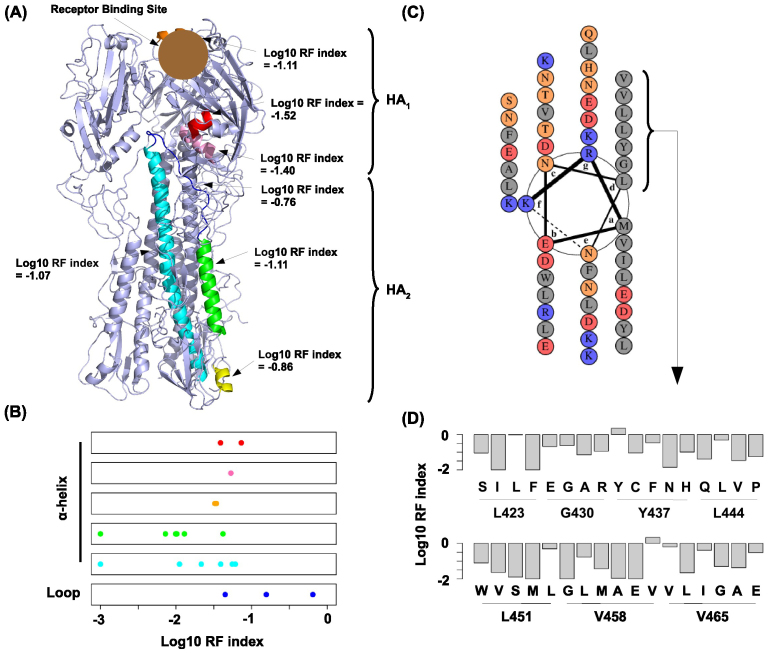
Structural analysis on hemagglutinin. (A) All *α*-helices (orange, red, pink, cyan, green, yellow) and a non-structural loop (blue) in HA are highlighted. Mean log_10_ RF indices for individual highlighted structural elements are shown. (B) The log_10_ RF indices for all observed X → P mutations (where X can be any amino acids but P) in individual highlighted structural elements are plotted as stripcharts. The colors of the stripcharts match the highlight colors of the corresponding structural elements in panel A. The bottom stripchart represents the non-structural loop that undergoes *α*-helix formation during membrane fusion. (C) Helical wheel was constructed by DrawCoil 1.0 (http://www.grigoryanlab.org/drawcoil/). Amino acid property of each residue is color coded. Polar: orange; Hydrophobic: grey; Positively charged: red; Negatively charged: blue. (D) The bar chart represents the RF indices of all profiled amino acid substitutions at heptad position d. RF indices of silent mutations are also included for comparison.

We further investigated each *α*-helix by computing their individual mean log_10_ RF index (Fig. 4A). As expected from the SASA analysis, the *α*-helices located at the core of HA_1_ are the least tolerant to mutations (red and pink, mean log_10_ RF index = −1.52 and −1.40 respectively). The other *α*-helix in HA_1_ is also relatively intolerable to mutations (orange, mean log_10_ RF index = −1.11), which is consistent with its role in receptor binding for viral entry^25^. In HA_2_, the two *α*-helices located at the stem-loop region are relatively intolerable to mutations (green and cyan, mean log_10_ RF index = −1.11 and −1.22 respectively), which can be attributed to their functional role in membrane fusion during viral entry^26^. In fact, all of the mean log_10_ RF indices reported above are lower than that of the entire HA (mean log_10_ RF index = −1.04). Together, these findings demonstrated that *α*-helices in HA are important for different functional mechanisms.

Interestingly, the non-structural loop region (blue) that interspaces the aforementioned helices (green and cyan) is more tolerant to mutations compared to its neighboring *α*-helices (mean log_10_ RF index = −0.76) (Fig. 4A). This region undergoes a transition from a non-structural loop to an *α*-helix during membrane fusion. Nonetheless, the relatively high RF index in this region suggests that the structural requirement for this transition is not stringent. This is further evidenced by a proline substitution analysis (Fig. 4B). Among all 20 standard amino acids, proline has the poorest *α*-helix formation propensity as its presence would result in a break or a kink of an *α*-helix^27^. Therefore, it is expected that proline substitutions in an *α*-helix would carry a low RF index (deleterious). Indeed, all proline substitutions in the HA *α*-helices have a log_10_ RF index < −1. In contrast, two out of three proline substitutions in the non-structural loop have a log_10_ RF index > −1 (−0.81 and −0.19 respectively). This result suggests that the formation of a continuous *α*-helix in this region is not a strict requirement during membrane fusion.

We also performed an in depth analysis on the *α*-helix that is important for homotrimer formation (colored in cyan in Fig. 4A). Helix wheel projection showed that high hydrophobicity was critical at heptad position d (Fig. 4C). We further investigated the RF index of those amino acid substitutions at heptad position d (Fig. 4D). Silent mutation at G430 had the lowest RF index (0.24) among all silent mutations at this heptad position. This RF index was employed as a reference to identify substitutions that has a relatively neutral fitness effect. Only three out of 27 amino acid substitutions at this heptad position has an RF index ≥0.24, namely Y437F (RF index = 0.35), V465I (RF index = 0.40) and V465A (RF index = 0.30). These three substitutions are conserved in volume and hydrophobicity, which suggests that residues at heptad position d has a stringent structural constraint in side chain conformation and hydrophobicity for homotrimer formation.

### Identification of essential regions

**Figure 5:**
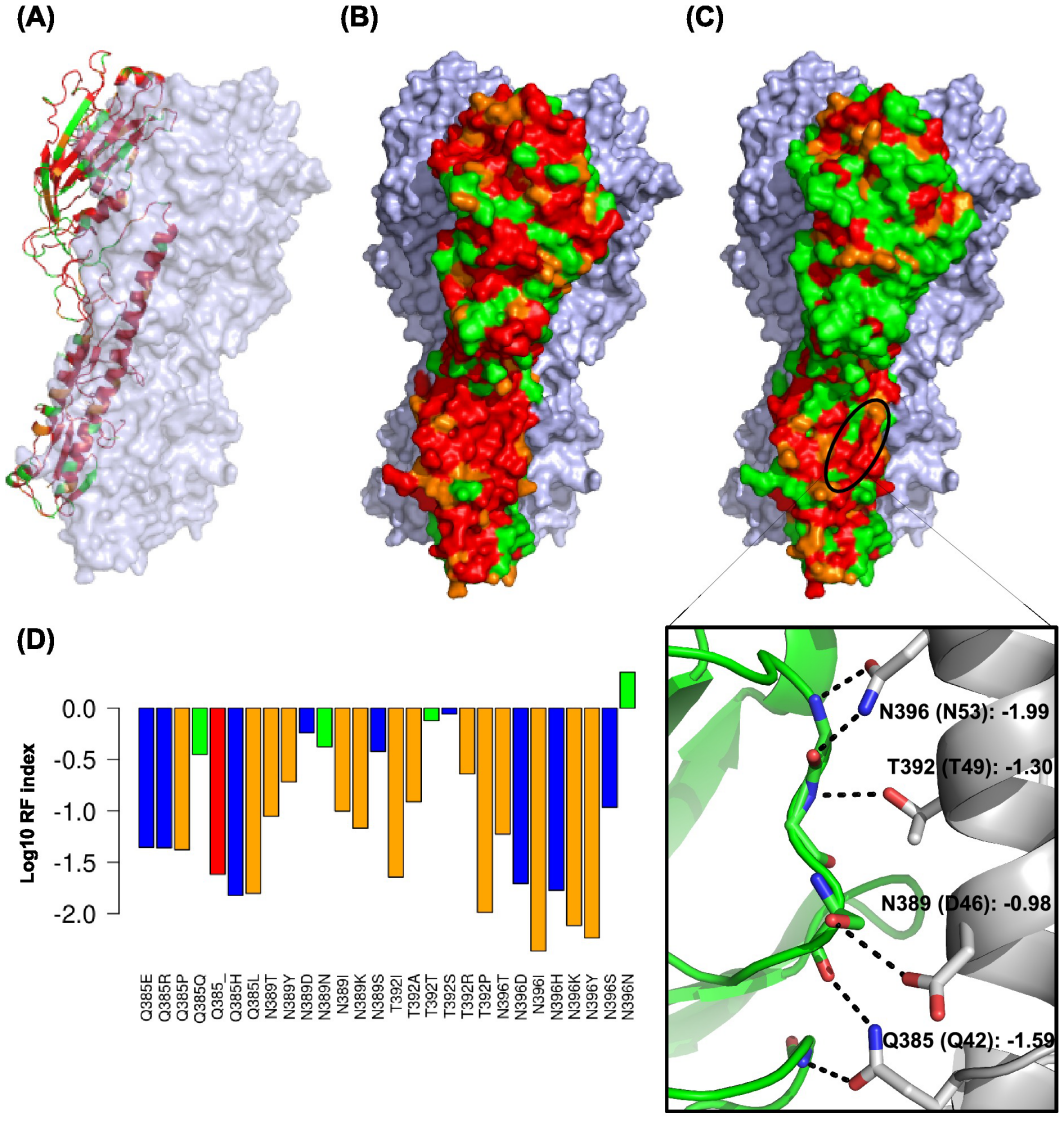
Essential regions on hemagglutinin. (A–B) The RF indices of the most destructive missense substitutions in the profiling data for individual amino acids are projected on the HA protein structure to identify essential regions intolerable to mutations. (C) The RF indices of the least destructive missense substitutions in the profiling data for individual amino acids are projected on the HA protein structure to identify essential regions intolerable to mutations. The inset represents the side chain interaction between HA (grey) and the proposed influenza universal antibody CR6261 (green) (PDB: 3GBN)^28^. Parentheses represent the residue naming according to HA_2_^28^. The mean log_10_ RF indices of nonconservative mutations for each residue are shown. Note that, residue 389 is an aspartic acid in the structure but is an asparagine in our wild type HA sequence. A compatible rotamer for T392 was generated using PyMOL to display the hydrogen bond. All hydrogen bonds (black dotted lines) are displayed as described^28^. (A–C) Red: RF index < 0.05; Orange: RF index < 0.1; Green: other. The structure is based on PDB: 1RUZ^49^. (D) The RF indices for missense mutations within the universal antibody recognition sites are shown. Types of amino acid substitution are color coded with red: nonsense substitution; orange: nonconservative substitution; blue: conservative substitution; green: silent mutation. A conservative substitution is defined as having a positive score in the blosum80 matrix.

Our profiling also provides information to identify possible essential protein surfaces and indispensable regions useful for vaccine epitopes. Our genetic platform provides the relative fitness effects of an average of five substitutions per amino acid residue. The RF indices of the most destructive substitutions in our dataset can be projected on the HA structure to identify putative functional regions that cannot tolerate certain amino acid substitutions (Fig. 5A–B). Whereas the RF indices of the least destructive substitutions for HA is projected on the HA structure to identify essential regions that are intolerable to any substitution (Fig. 5C). As expected, the trimer formation surface (Fig. 5A) and the stem domain (Fig. 5B–C), which is the major functional component of the membrane fusion machinery in HA, show as essential regions in our profiling data. In addition, our dataset identified the cross-subtype conserved influenza HA stalk region as an indispensable region (Fig. 5C–D), which is at the binding site of the proposed influenza universal antibody, CR6261^28^,^29^. The side-chain interactions at this site are important for CR6261 recognition. Although several missense substitutions in the binding site are allowed, they are conservative substitutions (N389D and T392S) unlikely to disrupt antibody recognition (Fig. 5C–D). It confirms the promising aspect of the proposed universal antibody^29^. In addition, the main antigenic sites on the globular head of HA were largely tolerable to substitutions (Fig. 5C). This observation suggests a functional basis for the tendency of this domain to rapidly undergo genetic drift, which adversely affects both natural and vaccine-induced immunity^30^. Overall, our work details the genetic cost for individual point mutations across HA – the primary target of anti-influenza neutralizing antibodies^28^,^29^,^30^,^31^,^32^. This dataset therefore provides a valuable reference for rational vaccine design.

## Discussion

Traditionally, critical residues on a viral genome are discovered by testing individual mutants and requires multiple assays to dissect the associated biological functions. The low throughput nature of this process limits the number of mutants tested. In this study, we have developed a comprehensive strategy using the influenza A virus as a model system to profile the fitness effects of individual point mutations and to identify essential residues throughout the HA gene in a high-throughput manner.

Recently, two studies that describe the development of a deep sequencing-based high-throughput genetic platform at single-nucleotide resolution have been reported in the literature^33^,^34^. Robins et al. probed for essential residues in T7 bacteriophage and T7-like virus JSF7 of *Vibrio cholerae* using mutant libraries constructed by chemical-induced transition of a GC base pair to an AT base pair^33^. Acevedo et al., on the other hand, interrogated the fitness effects of individual point mutations that naturally emerged in an evolving poliovirus population which has a high mutation rate, rather than employing any engineering strategy of introducing mutations^34^. In this study, we have developed a novel strategy which utilizes a saturated point mutation library together with a sensitive sequencing approach. When compared to the two aforementioned approaches, our method is more comprehensive and unbiased due to the mutant library construction strategy, which is independent of spontaneous mutations. This application can be extended to other influenza genes and to other genetically manipulable viruses under any applied selection condition at a single-nucleotide resolution level.

Identification of residues essential for viral replication is often inferred by sequence conservation. Observed sequence conservation derives from the viral sequences that initiated the endemic, and is influenced by the host genetic background and the specific immune responses associated with the host. Conservation is not equivalent to essentialness for viral replication in cells. Mutational analysis of conserved amino acid residues on influenza A virus has revealed that a significant fraction of conserved residues are dispensable in viral replication^35^,^36^,^37^. In addition, new mutations emerge every flu season, implying that a certain portion of residues that are conserved currently are still capable to mutate in the natural environment and provide a fitness advantage under future unforeseen selection pressures. This also suggests that a conserved amino acid may not necessarily be essential to viral replication. Additionally, analyses of conserved sequences provide information on viral genetic elements that survived in the selected human population in recent history, but does not provide much information on viral genetic elements that were unable to survive the selection process, nor about which host factor was responsible for exerting the selection. Our approach provides a complementary, yet more direct approach to identify amino acid residues that are critical for viral replication in a defined cellular environment. Nonetheless, to be more comprehensive, similar studies should be performed with strains across subtypes and include different selection conditions.

In summary, the platform described here enabled the simultaneous functional profiling of point mutations across the entire influenza HA at single-nucleotide resolution to determine their roles in viral replication. Our platform provides an efficient tool to address several important biomedical questions. The fitness profiling data allows the study of structure-function relationships at single-amino acid resolution. It enables the search for essential protein surfaces on available structures and thus offers a reference for drug design approaches that aim to increase the genetic barrier for the emergence of escape mutations^38^,^39^,^40^. Essential peptide stretches could also provide potential targets for drug and vaccine development^41^. Our genetic platform can be applied to study viral genome dynamics and identify critical residues for virus-host interactions in a specific cellular responses (such as apoptosis, autophagy, inflammasome induction, ER stress, etc.) and immune responses (such as NK cells, T cells, antibodies, macrophages, cytokines, etc.)^42^,^43^. The current development of a live attenuated influenza vaccine has been based on the modification of NS1 to increase interferon sensitivity^44^. However, this study provides a platform to explore alternative strategies. Comparing the *in vitro* fitness profile with an *in vivo* profile could also permit the identification of mutants that replicate efficiently *in vitro* but not *in vivo*. The resultant information when coupled with known mutants that are sensitive to a specified immune response could help achieve a higher titer during vaccine production, but exhibit an attenuated phenotype after injection into the human body where an intact immune system is present. Most importantly, our platform is applicable to other viral or microbial genomes where genetic manipulation is available in the laboratory. The sensitivity of our platform will increase as NGS technology improves. With the continued development of NGS technology, we foresee that our platform will be further advanced and can be applied at a much lower cost.

## Methods

### Viral mutant library and point mutations

The plasmid mutant library was created by performing error-prone PCR on the HA segment of the eight-plasmid reverse genetics system of influenza A/WSN/1933 (H1N1)^17^. We PCR-amplified the HA gene insert with error-prone polymerase Mutazyme II (Stratagene, La Jolla, CA). The mutation rate of the error-prone PCR was optimized by adjusting the input template amount to avoid the accumulation of deleterious mutations. The restriction enzyme site BsmBI was present in the PCR primers, and used to clone into a BsmBI-digested parental vector pHW2000. Ligations were carried out with high concentration T4 ligase (Life Technologies, Carlsbad, CA). Transformations were carried out with electrocompetent MegaX DH10B T1R cells (Life Technologies), and >200,000 colonies were scraped and directly processed for plasmid DNA purification (Qiagen Sciences, Germantown, MD). As extensive trans-complementation was expected during the transfection step, >35 million cells were used for transfection to average out any bias or artifact generated from possible trans-complementation. Point mutants for the validation experiment were constructed using the QuikChange XL Mutagenesis kit (Stratagene) according to the manufacturer's instructions.

### Transfections, infections, and titering

C227 cells, a dominant negative IRF-3 stably expressing cell line derived from human embryonic kidney (293T) cells, were transfected with Lipofectamine 2000 (Life Technologies) using the HA mutant library plasmid plus 7 other wildtype plasmids. Supernatant was replaced with fresh cell growth medium at 24 hrs and 48 hrs post-transfection. At 72 hrs post-transfection, supernatant containing infectious virus was harvested, filtered through a 0.45 um MCE filter, and stored at −80 degree Celsius. The TCID_50_ was measured on A549 cells (human lung carcinoma cells).

Virus from the C227 transfection was used to infect A549 cells at an MOI of 0.05. Infected cells were washed three times with PBS followed by the addition of fresh cell growth medium at 2 hrs post-infection. Virus was harvested at 24 hrs post-infection. For the mutant library profiling, HA mutant library was passaged for two 24-hour rounds in A549 cells. Our pilot experiments as well as our previous study revealed that two rounds of passaging were suffcient for profiling^45^. The biological duplicate was performed by an independenly transfected viral library, followed by two rounds of passaging as described above.

### Sequencing library preparation

Viral RNA was extracted from the passaged viral mutant library using QIAamp Viral RNA Mini Kit (Qiagen Sciences) and was reverse transcribed to cDNA using Superscript III reverse transcriptase (Life Technologies). DNA from the plasmid library or cDNA from the passaged viral mutant library were amplified with both forward and reverse primers each flanked with a 6 “N” tag and the Illumina flow cell adapter region. Flanking region for 5′ primer: 5′-CTA CAC GAC GCT CTT CCG ATC TNN NNN N-3′, Flanking region for 3′ primer: 5′-TGC TGA ACC GCT CTT CCG ATC TNN NNN N-3′. Following PCR, 12 amplicon products were pooled together. 1.5 million copies of the pooled product were used as the input for the second PCR, which was equivalent to 10 paired-end reads per molecule if 15 million paired-end reads were sequenced. 5′-AAT GAT ACG GCG ACC ACC GAG ATC TA CAC TCT TTC CCT ACA CGA CGC TCT TCC G-3′ and 5′-CAA GCA GAA GAC GGC ATA CGA GAT CGG TCT CGG CAT TCC TGC TGA ACC GCT CTT CCG-3′ were used as the primers for the second PCR. Products of the second PCR were submitted for next generation sequencing. The error-correction technique described in this study shared the same philosophy as described for detecting rare mutations in human cells^18^. However, this study included the fine restraint of limiting the input tagged template copy number and PCR efficiency during the second step PCR to accurately control the distribution of cluster size in the sequencing output to a median of 10. Raw sequencing data have been submitted to the NIH Short Read Archive under accession number: BioProject PRJNA243038.

### Data analysis

Sequencing reads were mapped by BWA with a maximum of six mismatches and no gap^46^. Amplicons with the same tag were collected to generate a read cluster. Since each read cluster was originated from the same template, true mutations were called only if the mutations occurred in 90% of the reads within a read cluster. We acknowledged that this error-correction approach would only correct errors that occured during the deep sequencing process but not those that were introduced during the reverse transcription process. Read clusters with a size below three reads were filtered out. Read clusters were further conflated into “error-free” reads. Average coverages in terms of “error-free” reads were 177028 per nucleotide in the plasmid mutant library, 112355 per nucleotide in replicate 1 of passaged viral mutant library, and 161773 per nucleotide in replicate 2 of passaged viral mutant library (Fig. S1A). Relative fitness index (RF index) for individual point mutations was computed by: 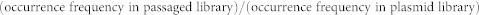
For all the downstream analysis, only point mutations covered with ≥30 tag-conflated reads (“error-free” reads) in the plasmid library were included. This arbitrary cutoff filtered out mutants with low statistical confidence, which is ~16% of all possible point mutations (Fig. S1B). In addition, all C → A and G → T mutations are not included in the reported dataset due to an observed DNA oxidative damage during library preparation^47^. The RF index presented in Table S1 was calculated by averaging all RF indices available for a given amino acid substitution.

### Structural analysis

The solvent accessible surface area (SASA) for individual residues was computed from PyMOL using the default “get area” function. SASA obtained from the folded structure was then normalized with the SASA calculated from an unfolded structure to obtain the relative SASA. Secondary structure assignment was performed by STRIDE^48^. The structural analysis was based on PDB: 1RUZ^49^. A two-tailed Student's t-test was employed to compare the log10 RF indices in different types of structural elements. Only missense mutations are included in the analysis unless otherwise stated.

## Author Contributions

N.C.W., A.P.Y. and R.S. designed the experiment, A.P.Y. created the plasmid library, N.C.W. conducted the experiments, R.G.C., S.F.N. and X.L. performed the sequencing, N.C.W. performed the data analysis, S.C., I.L. and C.L. assisted sequence mapping, L.Q.A., J.F., H.H.L. and N.N. provided experimental support, C.A.O., H.Q. and T.W. provided intellectual input. N.C.W., A.P.Y. and R.S. supervised the project, N.C.W. and R.S. wrote the text.

## Supplementary Material

Supplementary Information Supplemental Information

## Figures and Tables

**Table 1:** Comparison with phenotype reported in the literature

Substitution^a^	RF index	Expected Phenotype^b^
Y174H (Y159H)^c^	0.04	Deleterious
D238G (D225G)^d^	0.23	Deleterious
Y342H (Y328H)^e^	0.16	Deleterious
Y342C (Y328C)^e^	0.11	Deleterious
Y342N (Y328N)^e^	0.04	Deleterious
Y342F (Y328F)^e^	0.37	Neutral
D111E (D110E)^f^	1.03	Neutral
Q299R (Q298R)^f^	1.00	Neutral

^a^Positions of the substitutions are named based on our wild type protein sequence. Positions of substitutions in the parentheses represent the naming in the corresponding reference.

^b^Expected phenotype is classified into deleterious and neutral based on their reported phenotype.

^c^Temperature sensitive mutation, in which 37°C is a non-permissive temperature.

^d^Prefers *α*2,3 linked sialic acid receptor (avian) and does not efficiently bind to *α*2,6 linked sialic acid receptor (human).

^e^Only Y and F at this residue support efficient viral replication in our growth condition that is in the absence of trypsin.

^f^Mutations that were confirmed to thermodynamically stabilize the HA protein.
