# Supplementary material for: High-throughput profiling of influenza A virus hemagglutinin gene at single-nucleotide resolution
Source: Sci Rep. 2014 May 13;4:4942. doi: 10.1038/srep04942 (PMC4018626; doi:10.1038/srep04942)
Supplement: Supplementary Information — Supplemental Information [file srep04942-s1.pdf]

Supplemental Information

High-throughput profiling of influenza A virus hemagglutinin gene at single-nucleotide resolution

Nicholas C. Wu[1,2,7], Arthur P. Young[1,7], Laith Q. Al-Mawsawi[1], C. Anders Olson[1], Jun Feng[1], Hangfei Qi[1], Shu-Hwa Chen[3], I-Hsuan Lu[3], Chung-Yen Lin[3], Robert G. Chin[4], Harding H. Luan[1], Nguyen Nguyen[1], Stanley F. Nelson[2,4], Xinmin Li[5], Ting-ting Wu[1], Ren Sun[1,2,6,*]

[1]Department of Molecular and Medical Pharmacology, David Geffen School of Medicine, University of California, Los Angeles, CA 90095, USA

[2]Molecular Biology Institute, University of California, Los Angeles, CA 90095, USA

[3]Institute of Information Science, Academia Sinica, Taipei, Taiwan

[4]Department of Human Genetics, David Geffen School of Medicine, University of California, Los Angeles, CA 90095, USA

[5]Department of Pathology and Laboratory Medicine, David Geffen School of Medicine, University of California, Los Angeles, CA 90095, USA

[6]AIDS Institute, University of California, Los Angeles, CA 90095, USA

[7]These authors contributed equally to this work

*E-mail: RSun@mednet.ucla.edu

This file includes: Figure S1 and Table S1

(A)

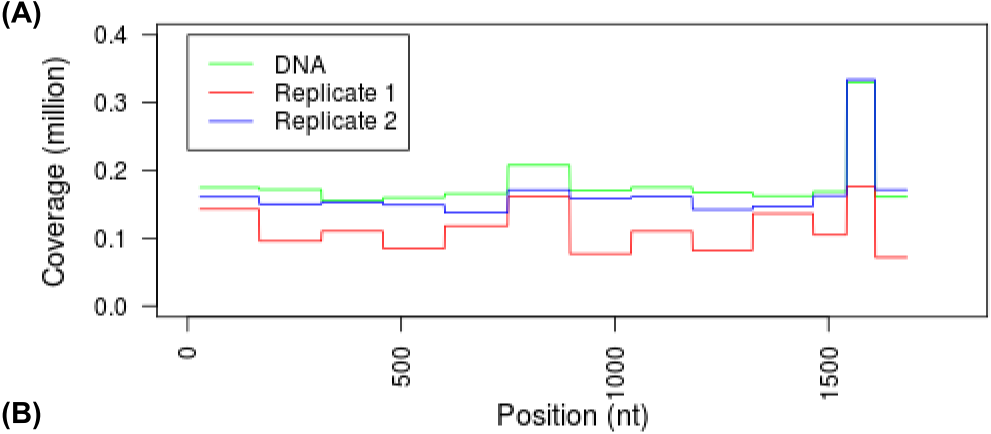

(B)

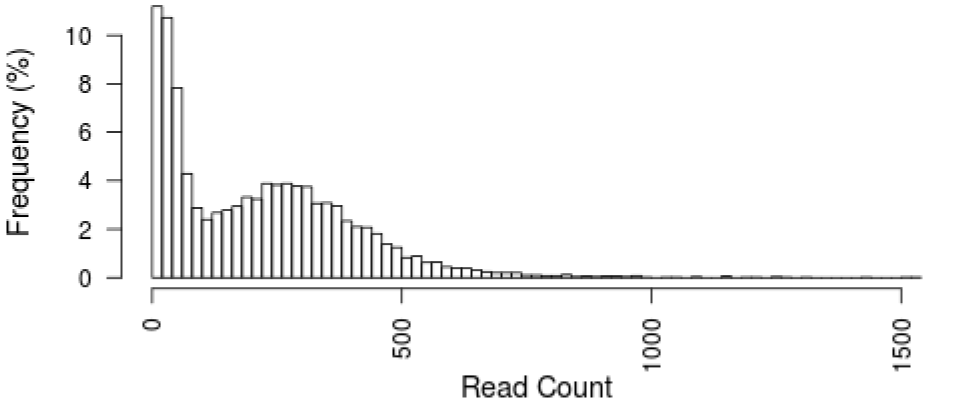

Supplemental Figure 1. Sequencing coverage and depth. This figure displays the sequencing depth in terms of number of ``error-free'' reads. In other words, it shows the number of ``error-free'' reads that cover a particular nucleotide or mutation. (A) Sequencing depths across the HA gene in plasmid mutant library (DNA), replicate 1 of passaged viral mutant library (Replicate 1) and replicate 2 of passaged viral mutant library (Replicate 2) are shown. (B) The distribution of ``error-free'' read counts of individual point mutations in the plasmid mutant library.

Table S1

Position	Substitution	RF Index
8	L8L	0.177970207
9	L9V	0
9	L9S	0.110407325
9	L9_	0.066555122
9	L9F	0.133090103
9	L9L	0.391744356
9	L9I	1.002017189
10	Y10_	0.051545186
10	Y10Y	0.946121557
10	Y10N	0.043307384
10	Y10H	0.333323406
10	Y10F	0.296502507
10	Y10C	1.185490814
11	A11P	0.021734426
11	A11T	3.170195808
11	A11V	3.969209344
11	A11A	1.542882593
11	A11G	0.009879285
12	F12V	0.892617789
12	F12S	0.112397918
12	F12Y	0.225241888
12	F12F	0.854762541
12	F12C	0.033960041
12	F12L	1.049767121
12	F12I	0.926398247
13	V13V	1.131490256
13	V13I	0.793180591
13	V13L	0.189852371
13	V13G	0.009702869
13	V13E	0.057972982
13	V13A	0.152468725
14	A14V	0.350644654
14	A14A	0.759078996
14	A14G	0.011560865
14	A14P	0.025078184
14	A14T	0.78723965
15	T15S	0.376438265
15	T15P	0.008265517
15	T15T	0.377137367
15	T15A	0.140823721
15	T15I	0.657579123
16	D16G	0.11967578
16	D16E	0.536138157
16	D16D	0.980437276
16	D16H	1.397283135
16	D16V	0
16	D16N	1.159090133
17	A17A	0.479198821
17	A17G	0.362664416
17	A17V	0.076663373

17	A17T	0.185288874
17	A17P	0.176415799
18	D18N	0.027106172
18	D18A	0
18	D18E	0.113252643
18	D18D	1.986060693
18	D18G	0.059136046
18	D18V	0
19	T19T	0.311304791
19	T19S	2.32777517
19	T19P	0.112247748
19	T19I	1.630211957
19	T19A	0.011960349
20	I20M	0.997602409
20	I20L	0.302223819
20	I20I	0.254006971
20	I20K	0.016166632
20	I20T	0.062419671
20	I20V	0.349632883
21	C21_	0.029002339
21	C21S	0.010756038
21	C21G	0.037258353
21	C21C	1.309661091
21	C21R	0.052002006
21	C21Y	0.037528957
22	I22V	0.180275845
22	I22T	0.045731977
22	I22M	0.10949626
22	I22L	0.021066206
22	I22K	0.006715168
22	I22I	0.960001337
23	G23R	0
23	G23S	0.010738353
23	G23G	1.030971722
23	G23D	0.056554272
23	G23A	0.035457871
24	Y24N	0.003996571
24	Y24Y	0.591320166
24	Y24_	0.025470031
24	Y24C	0.071876187
24	Y24F	0.224199756
24	Y24H	0.210317999
25	H25D	0.034682595
25	H25L	0.237965437
25	H25H	0.857584784
25	H25Q	0.409036315
25	H25P	0.198517162
25	H25R	0.047763048
25	H25Y	0.044844809
26	A26A	0.835435275
26	A26G	0
26	A26V	0.189047095

26	A26P	0.007874792
26	A26T	0.085801059
27	N27Y	0.052477488
27	N27S	0.041595392
27	N27K	0.025485345
27	N27N	0.373748174
27	N27D	0.024331288
27	N27I	0.006220624
28	N28T	0
28	N28S	0.034262426
28	N28Y	0.023571611
28	N28D	0.009935676
28	N28N	0.564606928
28	N28I	0.005402621
29	S29L	0.015623883
29	S29T	0.017127486
29	S29P	0.029810796
29	S29S	0.710734678
30	T30T	1.058899598
30	T30S	0.057788083
30	T30P	0.197130243
30	T30A	0.061149701
30	T30I	0.055952717
31	D31V	0.027777073
31	D31E	0.048171497
31	D31A	0.174889032
31	D31D	0.203843944
31	D31G	0.03032309
31	D31N	0.070348412
32	T32I	0.030059639
32	T32P	0.056194422
32	T32S	0.352869897
32	T32T	0.447538866
32	T32A	0.062801801
33	V33V	0.230666453
33	V33D	0.007921308
33	V33G	0.095112618
33	V33A	0.047098274
33	V33L	0
33	V33I	0.856363455
34	D34V	0
34	D34N	0.041442073
34	D34A	0.059457637
34	D34G	0.025343287
34	D34E	0.049706104
34	D34D	0.521608004
35	T35I	0.014574245
35	T35T	0.567359939
35	T35A	0.005615103
35	T35P	0
35	T35S	0.00509003
36	I36M	0.07933209

36	I36L	0.172546931
36	I36K	0.002517071
36	I36I	0.19565653
36	I36V	0.068161569
36	I36T	0.102315608
37	L37R	1.152654565
37	L37P	0.022062205
37	L37V	0.05021778
37	L37F	0.143412553
37	L37H	0.203625789
37	L37L	0.364895364
38	E38D	1.998139536
38	E38E	2.21171333
38	E38G	0.02802448
38	E38K	0.543351134
38	E38V	0.035618106
39	K39K	0.492002373
39	K39M	0.015354857
39	K39E	0.054859503
39	K39_	0.020061203
39	K39R	0.061065488
40	N40Y	0.022135392
40	N40S	0.110319395
40	N40T	0.078682994
40	N40K	0.051389583
40	N40I	0.012539092
40	N40N	0.090107398
40	N40D	0.297273683
41	V41V	0.416953402
41	V41M	0.021645784
41	V41L	0
41	V41E	0.023558782
41	V41G	0.043468853
41	V41A	0.021306084
42	A42T	0.194763224
42	A42V	0.517724594
42	A42A	0.577917906
42	A42G	0
42	A42P	0.02312173
43	V43V	1.414480686
43	V43G	0.027802195
43	V43E	0.004665851
43	V43A	0.037129918
43	V43L	0
43	V43M	0.03476202
44	T44A	0.087806138
44	T44I	0.02010896
44	T44P	0.010867213
44	T44R	0.036224044
44	T44S	0.011850028
44	T44T	0.886781868
45	H45R	0.036110059

45	H45L	0.004085419
45	H45Q	0.11329214
45	H45P	0.033960041
45	H45Y	0.023194785
45	H45D	0.081504099
45	H45H	0.843912601
46	S46T	0.028500446
46	S46S	0.71017067
46	S46P	0.025827746
46	S46F	0.049137182
46	S46C	0.025272589
47	V47V	0.473619469
47	V47I	1.282552459
47	V47L	0.031499169
47	V47A	0.090738602
47	V47G	0
47	V47D	0.007462896
48	N48D	0.182598639
48	N48I	0.01013952
48	N48N	0.384342906
48	N48S	2.528627758
48	N48T	0.462003576
48	N48Y	0.007443297
49	L49V	0.01973059
49	L49R	0
49	L49Q	0.023772632
49	L49P	0.022597007
49	L49L	0.285991831
50	L50F	0.028813723
50	L50L	0.23224136
50	L50H	0.003215152
50	L50R	0.065861898
50	L50V	0.125390922
50	L50P	0.014128552
51	E51K	0.046661508
51	E51V	0.395489633
51	E51A	0.156221441
51	E51E	2.275280289
51	E51D	0.080724635
51	E51G	0.028569126
52	D52G	1.921434641
52	D52E	5.463195723
52	D52D	1.020646165
52	D52V	1.486478066
52	D52N	0.102561127
53	S53C	0.211047808
53	S53G	0.392348222
53	S53N	0.816157802
53	S53S	1.233913964
53	S53R	1.215997351
53	S53T	0
54	H54Y	0.043094121

54	H54Q	0.014685423
54	H54R	0.134596292
54	H54L	0.038549247
54	H54H	1.23062387
55	N55Y	0.011438458
55	N55T	1.612994047
55	N55S	0.314261178
55	N55N	4.420899562
55	N55H	0.103160387
55	N55I	0.037891712
55	N55K	0.033358298
55	N55D	0.54043749
56	G56E	0.872086727
56	G56A	0.883597752
56	G56R	0.190376291
56	G56G	0.850223461
57	K57I	0.860605339
57	K57E	0.039180275
57	K57N	0.363590288
57	K57K	0.496742054
57	K57R	0.909936354
57	K57_	0.099482845
58	L58L	2.150701749
58	L58Q	0.047191852
58	L58V	0.031773494
58	L58P	0.077161478
58	L58R	0.047149591
59	C59C	2.091747243
59	C59S	0.007249916
59	C59R	0.059731573
59	C59Y	0.047715907
59	C59_	0.043621237
60	K60_	0.086082186
60	K60I	0.00542107
60	K60N	2.032276337
60	K60K	0.201867311
60	K60E	0.255476057
60	K60R	0.314004375
61	L61S	0.111545438
61	L61_	0.020916879
61	L61F	0.368287747
61	L61I	5.891152131
61	L61L	0.95029565
62	K62E	0.525187534
62	K62_	0.024262204
62	K62I	0.011853324
62	K62K	1.417565552
62	K62N	0.504624921
62	K62T	0.009224563
62	K62R	0.948302036
63	G63G	0.398617385
63	G63E	0.189176459

63	G63R	0.032840336
64	I64T	0.271373001
64	I64V	1.130641202
64	I64M	0.545872098
64	I64L	0.638934566
64	I64I	2.246980709
64	I64K	0.055881863
65	A65P	0.190606905
65	A65V	0.306966762
65	A65T	0.303073428
65	A65A	1.755592034
66	P66S	0.03627518
66	P66L	0.022102616
66	P66P	0.875476967
67	L67V	0.283189348
67	L67L	0.416687571
67	L67Q	0.020285679
67	L67P	0.086960314
68	Q68E	0.063546988
68	Q68H	0.92794696
68	Q68L	0.140617326
68	Q68P	0.031229045
68	Q68R	1.202657786
68	Q68_	0.07502095
68	Q68Q	0.851020064
69	L69M	0.031266294
69	L69L	0.631428012
69	L69F	0
69	L69_	0.056173581
69	L69S	0.022437787
69	L69W	0.0453264
70	G70E	0.742071504
70	G70R	0.405704327
70	G70G	0.033059127
71	K71_	0
71	K71T	0.389026692
71	K71R	0.218201176
71	K71N	0.598322083
71	K71I	1.340485698
71	K71E	0.697744327
71	K71K	0.117475226
72	C72_	0.12585399
72	C72Y	0.028951183
72	C72R	0.030957398
72	C72S	0.007191215
72	C72C	0.284430045
73	N73I	0.006220019
73	N73K	0.266454059
73	N73N	1.932833324
73	N73D	0.378368856
73	N73Y	0.011684899
73	N73S	0.367874245

73	N73T	0.955221011
74	I74T	0.079905515
74	I74V	0.158832062
74	I74F	0.261990182
74	I74M	0
74	I74N	0.014921999
74	I74I	0.241598467
75	T75P	0.07307194
75	T75A	0.035701525
75	T75I	0.09474209
75	T75S	0.146483907
75	T75T	0.70835902
76	G76R	0.050498281
76	G76E	0.011977443
76	G76A	0.478348091
76	G76G	0.265009143
77	W77C	0.012617496
77	W77R	0.006679577
77	W77_	0.034563005
78	L78F	0.045396234
78	L78H	0.024237314
78	L78P	0.038733659
78	L78L	0.605094114
79	L79L	6.645553085
79	L79M	0.060588114
79	L79V	0.076799096
79	L79S	0.047242297
79	L79_	0.02297775
79	L79W	0.063754867
80	G80E	0.06115688
80	G80G	0.226895509
80	G80R	0.054508051
81	N81N	0.172380541
81	N81K	0.006956233
81	N81S	0.120747234
81	N81Y	0
81	N81D	0.019938663
81	N81I	0.012334161
81	N81H	0.086654983
82	P82L	0.030065414
82	P82R	0
82	P82S	0.022938653
82	P82P	0.541357781
83	E83E	0.706884333
83	E83G	0.098123145
83	E83D	0.415832346
83	E83K	0.073310236
83	E83V	0.152491919
84	C84S	0.011836816
84	C84R	0.009078141
84	C84W	0.071642963
84	C84Y	0.027957431

84	C84C	1.410183159
85	D85V	0.087053697
85	D85N	0.062152921
85	D85G	0.067457278
85	D85E	0.014298072
85	D85D	1.153282039
86	S86S	0.425647118
86	S86T	0.475068663
86	S86L	4.202973769
86	S86P	0.822587864
87	L87L	0.549262941
87	L87V	0.228332011
87	L87Q	0.045551344
87	L87P	0.099674431
87	L87R	0
88	L88F	0.051084967
88	L88H	0.010582629
88	L88L	0.691135183
88	L88P	0.04702707
89	P89L	1.463350732
89	P89P	0.613957819
89	P89S	1.475139636
89	P89R	0.025996495
90	A90V	1.476906872
90	A90T	1.688679312
90	A90G	0.083334162
90	A90A	2.852189683
91	R91_	0.09283386
91	R91R	3.114530641
91	R91S	3.018191225
91	R91T	0.102402218
91	R91G	3.825359423
91	R91K	1.163127024
92	S92T	2.331043836
92	S92A	0.04079376
92	S92P	0.194962149
92	S92L	0.327533056
92	S92S	0.625877568
93	W93S	0.05599014
93	W93R	0.026220165
93	W93_	0.06187461
93	W93C	0
93	W93G	0.07386273
94	S94P	0.064409719
94	S94S	0.614782929
94	S94T	0.002723442
94	S94A	0.083575631
94	S94F	0.034983888
95	Y95N	0.037698456
95	Y95H	0.071086046
95	Y95F	0.007363814
95	Y95C	0.136203603

95	Y95_	0.090332431
95	Y95Y	1.144084969
96	I96T	0.029581014
96	I96V	0.60830779
96	I96S	0.053483119
96	I96F	0.016833091
96	I96L	0.108723343
96	I96M	0.15579865
96	I96N	0.023804178
96	I96I	0.58993221
97	V97V	3.415993408
97	V97A	0.712483551
97	V97G	0.019064096
97	V97E	0.020319198
97	V97L	0.006538994
97	V97I	1.632462356
98	E98G	0.034860424
98	E98D	0.059451366
98	E98E	1.487337833
98	E98V	0.183058699
98	E98K	0.078038548
99	T99A	0.879195592
99	T99I	0.346684278
99	T99T	1.247610664
99	T99R	0.415181311
99	T99S	2.007750822
100	P100P	6.071815491
100	P100R	0.29489774
100	P100S	0.692929036
100	P100L	1.265570301
101	N101S	0.512125256
101	N101N	0.250828385
101	N101T	0.174134686
101	N101Y	0.435155279
101	N101D	0.499657741
101	N101K	0.207809318
101	N101I	0.20073574
101	N101H	0.253130329
102	S102S	0.546838374
102	S102P	0.537596884
102	S102A	0.941768819
102	S102F	0.021906942
102	S102T	0.230564485
103	E103E	1.488806894
103	E103K	0.071598299
103	E103V	0.152906208
103	E103G	0.111999047
104	N104D	1.022688331
104	N104I	0.476357
104	N104K	0.186794049
104	N104N	1.289662948
104	N104S	0.740843798

104	N104Y	0.576603076
105	G105G	1.255241804
105	G105E	0.029002747
105	G105R	0.061991312
106	A106A	0.528883908
106	A106G	0.033050044
106	A106P	0.194356196
106	A106T	0.1087023
106	A106V	0.311878072
107	C107Y	0.065054158
107	C107_	0.029336865
107	C107R	0.040842312
107	C107S	0.014541979
107	C107W	0.02158872
107	C107C	0.828498576
108	Y108C	0.068050157
108	Y108F	0.006374432
108	Y108N	0
108	Y108Y	0.887494405
108	Y108_	0.025843058
108	Y108H	0.077333152
109	P109S	0.04605103
109	P109L	0.03292597
109	P109P	1.044104038
109	P109R	0.033641875
110	G110R	0.036331726
110	G110E	0.061913604
110	G110G	0.478896
111	D111G	0.13597708
111	D111N	0.404488572
111	D111D	0.112956524
111	D111E	1.036897901
111	D111V	0.330596307
112	L112H	0.053313741
112	L112L	3.023270244
112	L112F	0.855969444
112	L112P	0.091561863
113	I113T	0.904489116
113	I113N	1.761868799
113	I113I	0.959959395
113	I113F	1.952686643
113	I113V	0.872204314
113	I113L	13.52236756
114	D114A	0.039011165
114	D114N	0.540915403
114	D114H	0
114	D114V	0.011239655
114	D114G	0.162225656
114	D114E	0.368447482
114	D114D	0.186801318
115	Y115_	0.044742931
115	Y115Y	2.134944706

115	Y115N	0.273792358
115	Y115H	0.358932022
115	Y115F	1.416991605
115	Y115C	0.065562658
116	E116E	0.358006995
116	E116G	0.15257761
116	E116K	0.071530667
116	E116Q	0
116	E116V	0.083964622
117	E117E	0.052396625
117	E117G	0.046227212
117	E117D	0.003013738
117	E117K	0.093663296
117	E117V	0.005729708
118	L118R	0
118	L118Q	0.002498936
118	L118P	0.053141464
118	L118L	1.003531317
119	R119R	0.303984378
119	R119W	1.088269582
119	R119T	1.26069534
119	R119K	1.086732991
119	R119G	0.042812778
120	E120V	0.013905201
120	E120Q	0.009561648
120	E120K	0.03349535
120	E120E	0.148904617
120	E120G	0.041195305
120	E120D	0
121	Q121E	0
121	Q121H	0.28462648
121	Q121L	0.125504116
121	Q121R	0.051491959
121	Q121_	0.005678175
121	Q121Q	0.512119771
122	L122S	0.01496635
122	L122_	0.002989778
122	L122L	1.574482043
122	L122M	0.208772589
123	S123C	0.073211066
123	S123G	0.437552709
123	S123N	0.164609055
123	S123R	0.01656121
123	S123S	3.17166832
123	S123T	0.882385148
124	S124P	0.08376232
124	S124T	0.563750915
124	S124S	0.703870166
124	S124L	0.38105719
125	V125V	1.191515404
125	V125I	0.279750145
125	V125L	0.121534782

125	V125A	1.511736527
125	V125E	0.005319704
126	S126T	0.670848926
126	S126P	0.09902579
126	S126S	0.866977601
126	S126L	0.209531717
127	S127T	0.092876004
127	S127S	0.582406136
127	S127P	0.050261867
127	S127L	0.169061242
127	S127A	0.082611878
128	L128_	0.009022152
128	L128S	0.157686516
128	L128V	0.38535625
128	L128I	1.635874568
128	L128L	1.021067982
128	L128F	0.272134509
129	E129D	0.319482324
129	E129E	0.218703111
129	E129K	0.161678691
129	E129V	0.135617008
129	E129G	0.123752843
130	R130G	0.056023673
130	R130K	0.463322191
130	R130S	0
130	R130R	0.878247453
130	R130_	0.018293614
131	F131L	0.019118816
131	F131Y	0.28349725
131	F131S	0.004504599
131	F131V	0.056935213
131	F131I	0.007137568
131	F131F	1.17641052
132	E132V	0.269945547
132	E132K	0.089937834
132	E132D	0.063600658
132	E132E	0.674946154
132	E132A	0.666255431
132	E132G	0.027530626
133	I133T	0.064167056
133	I133V	0.043718529
133	I133I	0.997884675
133	I133K	0.021981242
133	I133M	1.211704697
133	I133L	1.815771045
134	F134I	0.01762669
134	F134L	0.313600574
134	F134F	0.40406831
134	F134Y	0.078645286
134	F134S	0.023812532
135	P135L	0.121038775
135	P135R	0.038440427

135	P135S	0.102678763
135	P135P	0.823058067
136	K136_	0.029674329
136	K136Q	0.697461892
136	K136T	5.298906474
136	K136K	0.684305918
136	K136E	3.104002787
136	K136R	0.94080386
136	K136M	2.463059318
137	E137E	0.731576349
137	E137D	0.677259113
137	E137K	0.097215714
137	E137G	0.17665266
137	E137V	0.19657741
138	S138N	0.29026114
138	S138G	0.892034533
138	S138C	0.017199696
138	S138T	0.014044019
138	S138R	0.19469724
138	S138S	0.665459662
139	S139L	0.080421639
139	S139T	0.52379442
139	S139P	0.039829354
139	S139S	0.489397925
140	W140_	0.07930739
140	W140R	0.011635609
140	W140G	0.04900434
140	W140C	0.016892245
141	P141P	0.751114296
141	P141S	0.044710981
141	P141R	0
141	P141L	0.230225133
141	P141A	0.043703455
142	N142D	0.052513916
142	N142I	0.021778085
142	N142S	0.014867006
142	N142T	0.061998374
142	N142Y	0.003619593
142	N142K	0
142	N142H	0.025231406
142	N142N	0.922916026
143	H143Q	0.029581649
143	H143Y	6.42954942
143	H143R	0.047107376
143	H143P	0.02324939
143	H143L	2.087895369
143	H143H	0.712498874
143	H143D	0.018005153
144	T144A	0.135886596
144	T144I	0.39858069
144	T144R	0
144	T144S	0.529498548

144	T144T	0.404599438
145	F145S	1.362034804
145	F145F	0.059084928
145	F145I	1.757014835
145	F145L	0.208895001
145	F145V	0.127326643
145	F145Y	0.592912477
146	N146N	1.987237711
146	N146K	0.056260552
146	N146H	0.129387406
146	N146I	0.006032945
146	N146D	2.951762293
146	N146Y	0.496388635
146	N146S	0.728708445
147	G147R	0.037871202
147	G147E	0.099989845
147	G147G	0.747136629
148	V148I	1.018149955
148	V148V	0.620701878
148	V148L	0.034541951
148	V148A	0.085756882
148	V148E	0.088280211
149	T149A	0.019496587
149	T149I	0.052061949
149	T149T	2.277631423
149	T149P	0.037956809
149	T149S	0.699158116
150	V150V	1.324632769
150	V150I	0.648850726
150	V150L	0.568626387
150	V150A	0.290189417
150	V150E	0.026484063
151	S151L	0.094600353
151	S151T	0.021431001
151	S151S	0.32923637
151	S151P	0.064070495
152	C152C	0.693410207
152	C152Y	0.155848228
152	C152R	0.039599908
152	C152S	0.02397466
152	C152W	0.007048967
153	S153P	3.729478791
153	S153T	0.775080753
153	S153A	0.017281338
153	S153F	0.077822422
153	S153S	1.631119286
154	H154Y	0.734619494
154	H154R	0.286360055
154	H154Q	0.310563297
154	H154P	0.173641477
154	H154L	0.801260582
154	H154H	0.675536697

155	R155R	0.305103744
155	R155S	1.767877912
155	R155W	0.35859429
155	R155T	0.085435667
155	R155K	5.379928103
155	R155G	4.670928538
156	G156R	0.386216479
156	G156E	1.4075061
156	G156G	1.707183504
157	K157N	2.260393025
157	K157I	3.449270308
157	K157K	0.325062185
157	K157E	11.44677781
157	K157_	0.018058514
157	K157R	0.670258017
158	S158C	0.026150767
158	S158R	0.092917199
158	S158S	0.832114281
158	S158N	0.903001966
158	S158G	0.226354865
159	S159N	0.640962515
159	S159R	0.073831822
159	S159G	0.976898934
159	S159C	0.030056267
159	S159S	0.635468612
160	F160C	0.052509079
160	F160F	0.576425182
160	F160I	0.023379166
160	F160L	0.073615478
160	F160S	0.134652146
160	F160Y	0.052184924
161	Y161F	0.372508897
161	Y161_	0.046210444
161	Y161N	0.025095205
161	Y161H	0.026982249
161	Y161C	0.029259661
161	Y161Y	0.794157404
161	Y161S	0
162	R162R	1.555796445
162	R162S	0.596788962
162	R162_	0.04144158
162	R162G	0.107847965
162	R162K	0.30860217
163	N163S	0.03757265
163	N163Y	0.027669072
163	N163D	0.103391813
163	N163N	0.890589898
163	N163K	0.016111924
163	N163I	0
164	L164L	0.923894319
164	L164M	0.132615889
164	L164F	0

164	L164_	0.005495136
164	L164S	0.038911211
164	L164V	0.169243988
165	L165L	0.668745155
165	L165V	0.208215517
165	L165Q	0.431872929
165	L165P	0.050711811
166	W166_	0.117351799
166	W166R	0.02396061
166	W166G	0.034439184
166	W166C	0
167	L167L	0.712709426
167	L167Q	0.033167719
167	L167P	0.047627655
167	L167R	0.039771284
167	L167V	0.40899166
168	T168A	0.079097501
168	T168M	0.134587169
168	T168T	0.583413763
168	T168P	0.020547031
168	T168R	0.126037898
168	T168S	0.074775137
169	K169E	0.029640087
169	K169M	0.074317458
169	K169T	0.448952628
169	K169R	1.186748864
169	K169_	0.027824123
169	K169K	1.006044969
170	K170K	0.845990476
170	K170M	0.812029107
170	K170E	0.124092456
170	K170_	0
170	K170R	0.41259652
170	K170T	0.009920768
171	G171E	1.091151508
171	G171G	0.568916768
171	G171R	0.039142332
172	D172V	0
172	D172G	0.07277428
172	D172E	0.05046295
172	D172D	0.90417975
172	D172H	0
172	D172N	0.072937484
173	S173T	0.799529591
173	S173P	0.160167838
173	S173S	0.504596365
173	S173L	1.538233165
174	Y174Y	1.121123933
174	Y174_	0.015002594
174	Y174S	0.043555438
174	Y174H	0.043473371
174	Y174C	0.094376799

174	Y174F	1.089693327
174	Y174N	0.009386168
175	P175S	0.534939936
175	P175L	0.33107434
175	P175A	0.018473154
175	P175P	0.816232648
175	P175R	0
176	K176E	0.039076197
176	K176M	0.335514334
176	K176K	1.642334956
176	K176_	0.018312913
176	K176T	0.363397192
176	K176Q	0.027709731
176	K176R	1.219106878
176	K176N	0
177	L177P	0.009545149
177	L177Q	0.045316412
177	L177R	0.060978286
177	L177L	4.562080048
178	T178T	1.025314669
178	T178P	0.011160864
178	T178S	0.185594542
178	T178I	0.587872612
178	T178A	0.308468868
179	N179K	0.586675862
179	N179I	0.749698578
179	N179Y	0.331636466
179	N179N	0.218640695
179	N179H	0.110630581
179	N179T	0.225205397
179	N179S	1.456221772
179	N179D	1.136504732
180	S180T	0.297767474
180	S180P	0.101923252
180	S180S	0.49569424
180	S180F	0.498684052
181	Y181_	0.017265931
181	Y181Y	0.404539028
181	Y181N	0.003780654
181	Y181H	0.192460809
181	Y181F	1.751236227
181	Y181C	0.069150079
182	V182V	0.82214321
182	V182G	0.062429847
182	V182E	1.217948114
182	V182A	0.659538171
182	V182L	0.251461098
182	V182M	0.587516082
183	N183Y	0.018240965
183	N183T	0
183	N183S	0.033371737
183	N183N	0.962797527

183	N183I	0.001483993
183	N183H	0
183	N183K	0
183	N183D	0.04104069
184	N184I	0.933555389
184	N184K	0.046788626
184	N184N	11.24872262
184	N184D	1.447433191
184	N184Y	0.131627625
184	N184S	2.074138579
185	K185K	0.498285618
185	K185R	0.18695395
185	K185E	6.042546425
185	K185I	1.079516888
185	K185N	0.908236207
185	K185T	0.030988965
185	K185_	0
186	G186R	0.382657636
186	G186G	2.345398747
186	G186E	1.227578635
187	K187K	0.676143531
187	K187N	0.460870826
187	K187I	0.054085228
187	K187E	0.336938878
187	K187_	0.021615435
187	K187R	1.242715125
187	K187T	7.091677232
188	E188V	1.015089635
188	E188K	3.988007195
188	E188G	0.136187269
188	E188E	0.528497714
188	E188D	1.25867663
189	V189I	4.888122831
189	V189A	0.170183041
189	V189D	0.024467601
189	V189V	0.763463263
190	L190F	0.245420091
190	L190H	0.032403789
190	L190L	0.418537825
190	L190R	0.190946207
190	L190P	0.143076283
191	V191V	1.003097095
191	V191E	0
191	V191A	0.127125324
191	V191L	0.187764419
191	V191I	1.307932857
192	L192L	1.479372711
192	L192V	0.382885549
192	L192Q	0.03400348
192	L192P	0.059304675
193	W193_	0.050392311
193	W193G	0.030851769

193	W193R	0.02223617
194	G194D	0.035870154
194	G194G	0.216746782
194	G194A	2.152844485
194	G194R	0
194	G194S	0.033898182
195	V195L	0.135774569
195	V195G	0.071354362
195	V195V	0.763837272
195	V195D	0.06893159
195	V195I	1.102343034
195	V195A	0.127507787
196	H196Q	0.028922624
196	H196P	0.053511298
196	H196R	0.029941459
196	H196Y	0.069526675
196	H196L	0.002349656
196	H196H	2.833949956
197	H197R	0.141606157
197	H197P	0.024917278
197	H197Q	0.630658814
197	H197D	0.396979685
197	H197L	1.696658793
197	H197Y	0.048026061
197	H197H	0.543315043
198	P198S	0.167369998
198	P198P	0.301490203
198	P198L	0.031250375
199	S199C	0
199	S199F	0.138132589
199	S199S	0.897090208
199	S199P	0.063176432
199	S199T	0.080120859
200	S200S	0.994676409
200	S200N	0.053844454
200	S200G	0.181004685
200	S200C	0.05340133
200	S200T	0.009872826
200	S200R	0.037375869
201	S201T	0
201	S201C	0.023510647
201	S201G	0.305192959
201	S201S	0.862683534
201	S201R	0.225080851
201	S201N	1.226801686
202	D202A	0.059567791
202	D202E	0.139663539
202	D202G	0.061992309
202	D202N	0.113990482
202	D202V	0.212604286
202	D202D	0.177826869
203	E203V	0.002988796

203	E203G	0.063684991
203	E203E	0.464764174
203	E203K	0.040573661
204	Q204H	0.179084878
204	Q204L	0.020058956
204	Q204R	0.009483822
204	Q204P	0.032598172
204	Q204Q	0.236631824
204	Q204E	0.028450288
204	Q204_	0.036531444
205	Q205E	1.687989449
205	Q205L	0.643626725
205	Q205Q	1.286747516
205	Q205P	0.034228511
205	Q205R	0.121884697
205	Q205_	0.025629324
206	S206C	0.02629952
206	S206N	0.698662843
206	S206G	0.3870538
206	S206R	0.073311405
206	S206S	0.364542748
206	S206T	0.082551115
207	L207L	1.75779886
207	L207P	0.052760048
207	L207F	0.029278998
207	L207H	0.007275455
207	L207R	0.116028017
208	Y208Y	0.184387001
208	Y208H	0.12227483
208	Y208F	0.007139211
208	Y208C	0.003326344
208	Y208_	0.005883239
208	Y208N	0.004379945
209	S209S	3.73714342
209	S209R	0.154033697
209	S209N	0.14626041
209	S209C	0.319106365
209	S209G	3.44714683
210	N210T	0.148821457
210	N210S	4.235376565
210	N210Y	0.244260617
210	N210D	2.605315842
210	N210N	0.324548843
210	N210I	0.744920672
210	N210K	0.39209736
211	G211R	0.153988117
211	G211G	0.904839089
211	G211E	0.513191847
212	N212I	0.965787189
212	N212T	0.03115047
212	N212N	1.654118057
212	N212K	0.955078724

212	N212H	0.628573964
212	N212D	0.642146986
212	N212Y	2.090705674
212	N212S	0.066249385
213	A213P	10.69404683
213	A213V	0.026196772
213	A213T	3.703131421
213	A213A	0.591419433
214	Y214_	0.020763167
214	Y214H	0.521621512
214	Y214N	0.012598327
214	Y214C	0.104599508
214	Y214F	1.605352326
214	Y214D	0.097399438
214	Y214Y	0.928020735
215	V215I	0.536340902
215	V215V	0.544048067
215	V215G	0.020074747
215	V215D	0
215	V215A	0.054626246
216	S216T	0.299359346
216	S216S	0.353395015
216	S216F	0.05817641
216	S216P	0.045704848
216	S216A	0.415688212
217	V217V	0.303949158
217	V217I	0.522220595
217	V217L	0.020074747
217	V217E	0.021229322
217	V217G	0.018820075
217	V217A	0.055158971
218	A218P	0
218	A218V	0.55356869
218	A218T	1.323547253
218	A218A	1.047197102
219	S219T	0.064870986
219	S219S	1.380495748
219	S219P	0.077425892
219	S219F	0.0688474
220	S220T	0.433925073
220	S220S	0.883808434
220	S220P	2.171369379
220	S220L	2.027514594
221	N221Y	0.123861287
221	N221S	0.168353511
221	N221I	0.257050348
221	N221K	0.146671408
221	N221N	1.275711937
221	N221D	0.551900498
222	Y222N	0.407549913
222	Y222H	0.316865308
222	Y222F	1.23894851

222	Y222C	0.264774265
222	Y222_	0.045384983
222	Y222Y	0.370150838
223	N223S	0.186513592
223	N223I	1.356403021
223	N223T	0.343464893
223	N223Y	0.123999985
223	N223D	0.553630521
223	N223K	0.687471308
223	N223N	0.759699437
224	R224K	0.786728951
224	R224G	1.891969581
224	R224W	0.362507731
224	R224R	0.165331224
225	R225K	0.973933052
225	R225G	0.095865265
225	R225_	0.044062164
225	R225R	0.127122183
225	R225S	0.01101663
226	F226F	0.019699518
226	F226L	1.086075126
226	F226I	0.139534356
226	F226S	0.052926885
226	F226Y	0.025073695
227	T227S	0.074010421
227	T227T	0.227360296
227	T227I	0.352975916
227	T227A	0.544920056
228	P228S	0.041683722
228	P228R	0.256501928
228	P228P	0.504748831
228	P228L	0.1112763
228	P228A	1.226973039
229	E229K	0.723316576
229	E229V	1.560798621
229	E229G	1.127243292
229	E229E	0.525266045
229	E229D	1.104528879
230	I230I	1.718114936
230	I230T	0.138058197
230	I230V	0.868174381
230	I230M	0.882090821
230	I230L	0.219742055
230	I230K	0.051719829
231	A231V	3.039618052
231	A231P	0
231	A231A	1.486009437
231	A231T	0.792326158
232	A232T	0.206352926
232	A232P	1.252488748
232	A232V	0.38363692
232	A232A	0.354565308

232	A232G	0.033426856
233	R233K	0.110596883
233	R233G	0.099282966
233	R233W	0.063559681
233	R233S	0.012546717
233	R233R	1.33715246
234	P234L	0.743887324
234	P234P	1.360004442
234	P234S	0.109750117
235	K235_	0.004620786
235	K235R	0.758950904
235	K235E	0.115333321
235	K235N	0.099559395
235	K235I	0.907437652
235	K235K	0.860710986
235	K235Q	0.045838229
236	V236V	0.565672905
236	V236I	0.542544868
236	V236L	7.877408895
236	V236A	0.11996352
236	V236E	0.027773698
237	R237_	0.032591942
237	R237S	1.129688278
237	R237R	1.917539783
237	R237G	0.186706296
237	R237K	0.810733958
238	D238G	0.230606628
238	D238N	0.040858497
238	D238D	0.450392874
238	D238E	0.338902629
238	D238V	0.010769318
239	Q239_	0.030869139
239	Q239R	0.068781419
239	Q239P	0
239	Q239Q	1.371585967
239	Q239L	0.013582572
239	Q239H	0.021387018
240	H240L	0.054016466
240	H240H	0.60926488
240	H240D	0.036595931
240	H240Y	1.063994936
240	H240R	0.03878179
240	H240Q	0.050919042
241	G241R	0.038228741
241	G241A	0.017114256
241	G241G	0.954943741
241	G241E	0.079701316
242	R242K	0.097580346
242	R242G	0.115554996
242	R242R	5.097331512
242	R242W	0.106216763
242	R242T	0.009482223

243	M243K	0.052890454
243	M243V	0.141842897
243	M243T	0.065698362
243	M243I	0.666788501
243	M243L	0.049140054
244	N244N	0.466882235
244	N244D	0.378942175
244	N244I	0.311155004
244	N244K	0.192888284
244	N244S	0.277337863
244	N244Y	0.715752945
245	Y245N	0.017372377
245	Y245H	0.078339681
245	Y245_	0.058002562
245	Y245Y	0.775548974
245	Y245F	0.00734442
245	Y245C	0.032569268
246	Y246F	1.176080228
246	Y246C	0.069312272
246	Y246H	0.394122844
246	Y246Y	0.27376473
246	Y246N	0.012994157
246	Y246_	0
247	W247_	0.040155617
247	W247R	0.023707604
247	W247G	0.035186164
248	T248A	0.193620069
248	T248I	0.573250406
248	T248S	3.701735342
248	T248T	0.166428065
249	L249S	0.166596104
249	L249_	0.018203096
249	L249F	0.562980782
249	L249M	2.365652075
249	L249L	0.46260018
250	L250P	0.042407041
250	L250Q	0.025420414
250	L250V	0.542346837
250	L250L	1.673177154
251	E251K	0.905045897
251	E251D	1.406768502
251	E251E	1.243645429
251	E251G	1.328316081
251	E251V	0.157117373
252	P252S	0.235545737
252	P252A	0.064962317
252	P252L	0.036380251
252	P252P	1.090733372
253	G253R	0.30652126
253	G253E	0.057817765
253	G253G	0.582083002
254	D254N	0.2783846

254	D254D	0.359797308
254	D254V	0.018405393
254	D254G	0.331067281
255	T255T	0.431126623
255	T255I	0.223709847
255	T255P	0.124635972
255	T255S	1.173412079
255	T255R	0
255	T255A	0.424056206
256	I256T	0.063173313
256	I256V	0.33272105
256	I256I	0.41989586
256	I256K	0.038486192
256	I256M	0.231595468
256	I256L	0.259630432
257	I257V	0.324571299
257	I257T	3.170015688
257	I257K	0.042642338
257	I257I	0.284839431
257	I257M	0.14192687
257	I257L	0.585238942
258	F258S	0.013695269
258	F258Y	0.416619486
258	F258F	0.814994022
258	F258I	0.188408542
258	F258L	1.002126321
259	E259V	0.044772375
259	E259K	0.3220779
259	E259E	0.428646605
259	E259G	0.112196968
260	A260T	0.147819517
260	A260V	0.025737481
260	A260A	0.434591163
260	A260G	1.414602753
261	T261I	0.114487062
261	T261A	0.140080781
261	T261S	0.398711413
261	T261P	0.062900614
261	T261T	0.322928765
262	G262R	0
262	G262D	0.043694135
262	G262G	2.906094898
262	G262A	0.092569632
262	G262S	0.055969
263	N263Y	0.002442164
263	N263T	0.042513451
263	N263S	0.028363798
263	N263N	0.585748507
263	N263K	0
263	N263H	0.022073319
263	N263I	0.003895657
263	N263D	0.026905283

264	L264V	0.187022189
264	L264Q	0.006614818
264	L264P	0.020257108
264	L264L	0.625693456
265	I265L	0.145972272
265	I265V	0.84831162
265	I265T	0.034134236
265	I265K	0.003317029
265	I265I	0.24768728
265	I265M	0.082916952
266	A266G	0.121942043
266	A266A	0.240603682
266	A266V	0.48443797
266	A266T	0.200193815
267	P267P	0.739230176
267	P267S	0.028000033
267	P267A	0.036788866
267	P267L	0.047176526
268	W268_	0.025722855
268	W268R	0.193504036
268	W268G	0.058409726
268	W268C	0.197692967
268	W268S	1.718653787
269	Y269H	0.161326675
269	Y269N	0.117852899
269	Y269F	0.886290007
269	Y269D	0.040467752
269	Y269C	0.054232768
269	Y269_	0.049668314
269	Y269Y	0.323919866
270	A270T	0.334387533
270	A270V	0.025746476
270	A270G	0.575033066
270	A270A	0.507168848
271	F271Y	0.228495997
271	F271V	0.032236565
271	F271S	0.026118462
271	F271I	0.010654376
271	F271L	0.100342722
271	F271F	0.30565876
272	A272A	0.472863948
272	A272V	0.928413931
272	A272P	0.007883328
272	A272T	0.65073941
273	L273V	0.517141961
273	L273L	0.496864091
273	L273Q	0.01566374
273	L273P	0.086865496
273	L273R	0.027930381
274	S274G	0.379567861
274	S274C	0.164749835
274	S274N	0.456890049

274	S274T	0.344189349
274	S274S	0.427158345
274	S274R	0.162417634
275	R275K	0.234517408
275	R275_	0.004633712
275	R275G	0.066752617
275	R275S	0.238233984
275	R275R	0.212690498
276	G276E	0.191410876
276	G276R	0.677505718
276	G276G	0.416691854
277	F277S	0.653729903
277	F277Y	0.218978859
277	F277V	0.178712705
277	F277F	0.604931613
277	F277I	1.025616096
277	F277L	0.427905421
278	E278K	3.436866467
278	E278E	0.449137077
278	E278D	0.347957065
278	E278G	0.221276647
278	E278V	0.623573709
279	S279T	0.197033943
279	S279P	0.050701582
279	S279S	0.098388605
279	S279F	0.015786598
279	S279C	0.025292345
280	G280A	0.218264156
280	G280D	0.142796497
280	G280G	0.364517114
280	G280S	0.221542966
280	G280R	0
281	I281N	0.007169484
281	I281I	0.831349206
281	I281T	0.03585604
281	I281S	0
281	I281F	0.112895883
281	I281V	0.596574027
282	I282V	0.992180879
282	I282T	0.060390734
282	I282S	0.062393351
282	I282F	0.769807776
282	I282L	0.425892215
282	I282M	0
282	I282N	0.009199989
282	I282I	0.249522506
283	T283I	1.713642256
283	T283S	0.695985965
283	T283A	0.370300527
283	T283P	0.024280651
283	T283T	0.188131126
284	S284S	0.424995357

284	S284T	0.586651374
284	S284L	0
284	S284P	0.09490406
284	S284A	2.274488816
285	N285K	0.353779633
285	N285H	0.018394433
285	N285I	0.34066041
285	N285N	0.43084421
285	N285D	0.357708651
285	N285Y	0.120086695
285	N285S	0.386737539
285	N285T	0.212567254
286	A286P	0
286	A286V	0.509688887
286	A286T	0.662015462
286	A286A	0.302345617
287	S287S	1.836720837
287	S287P	0.362852866
287	S287T	0.111434838
287	S287L	0.056353059
288	M288K	0.22933918
288	M288I	2.226016624
288	M288L	1.775888226
288	M288V	2.470530239
288	M288T	0.270486193
289	H289D	0.184619219
289	H289L	0.159913413
289	H289H	0.362181991
289	H289Q	0.717973302
289	H289Y	0.955857502
289	H289R	0.425553426
290	E290K	0.144852942
290	E290A	0.044974664
290	E290G	0.660737063
290	E290E	0.577972194
290	E290D	0.092473141
290	E290V	0.214077674
291	C291_	0.022394745
291	C291Y	0.101611501
291	C291R	0.01801147
291	C291S	0.000954428
291	C291G	0.009210447
291	C291C	0.511641177
291	C291W	0.041724919
292	N292D	0.317095887
292	N292N	0.935738893
292	N292K	0.081713731
292	N292H	0.045335704
292	N292I	1.089194437
292	N292T	0.021550896
292	N292S	0.714283341
292	N292Y	0.033932655

293	T293S	0.079375529
293	T293R	0
293	T293T	0.775178866
293	T293M	0.056734583
293	T293A	0.120074774
293	T293P	0.01556452
294	K294T	0.135087234
294	K294_	0.024074014
294	K294Q	0.195086833
294	K294R	0.231954843
294	K294N	0.449260157
294	K294K	2.048934574
294	K294M	0.836610137
294	K294E	1.262403128
295	C295C	1.47087083
295	C295R	0.026432315
295	C295S	0.008631316
295	C295Y	0.053446884
295	C295_	0.022452871
296	Q296_	0.025926232
296	Q296R	0.087458767
296	Q296H	0.006029963
296	Q296Q	0.725695563
296	Q296L	0.016141572
297	T297A	0.196421574
297	T297I	0.049883266
297	T297T	0.448247941
297	T297S	0.827539757
297	T297R	0.044406106
298	P298L	0.126368224
298	P298R	0
298	P298S	0.166764644
298	P298P	1.236617572
298	P298A	0.078706547
299	Q299H	0.121083847
299	Q299L	0.507517819
299	Q299P	2.637309126
299	Q299Q	2.456523678
299	Q299R	1.000449778
299	Q299_	0.067214696
300	G300R	0.0289041
300	G300G	1.027452366
300	G300E	0.02076744
301	A301T	0.055646788
301	A301P	0.018075117
301	A301A	1.145625817
301	A301V	0.069088162
301	A301G	0.055878317
302	I302L	0.60235389
302	I302T	0.295314479
302	I302I	0.174503164
302	I302M	1.117789241

302	I302V	0.713166365
302	I302K	0.014472754
303	N303Y	1.475805169
303	N303D	1.233167146
303	N303K	0.117999076
303	N303I	0.538235369
303	N303H	2.137378984
303	N303N	0.725682127
303	N303S	1.103266599
304	S304T	0.06917484
304	S304S	0.212189018
304	S304R	0.062310538
304	S304N	0.798963568
304	S304G	0.313217133
304	S304C	0.028007346
305	N305D	0.89066895
305	N305Y	0.097750196
305	N305S	2.04631284
305	N305H	0.14554132
305	N305K	0.254039946
305	N305N	1.322366706
305	N305I	0.188256629
306	L306L	0.034810867
306	L306H	1.15678371
306	L306F	0.127453373
306	L306P	0.131789959
307	P307P	1.800770156
307	P307A	0
307	P307S	0.098156117
307	P307L	0.045527385
307	P307R	0
308	F308F	0.387701957
308	F308L	0.066793522
308	F308I	0.001374945
308	F308V	0.17752574
308	F308S	0.124097008
308	F308Y	0.353111563
309	Q309_	0.056983557
309	Q309R	0.115416561
309	Q309P	0.110189165
309	Q309Q	1.285374685
309	Q309L	0.052470648
309	Q309H	0.010514284
309	Q309E	0
310	N310D	0.047246707
310	N310I	0.004563961
310	N310Y	0.010905554
310	N310T	0
310	N310S	0.46309589
310	N310N	0.530009841
310	N310K	0.058042527
310	N310H	0.08593198

311	I311V	0.342010893
311	I311K	0.038587443
311	I311I	0.44935717
311	I311M	0.137967706
311	I311L	0.100316813
311	I311T	0.150809141
312	H312D	0.387653576
312	H312H	0.479948051
312	H312L	0.013144207
312	H312R	0.056971169
312	H312Q	0.011657141
312	H312P	0.40624129
312	H312Y	0.305948941
313	P313P	0.587150668
313	P313S	1.191450251
313	P313A	0
313	P313L	0.073555389
314	V314V	0.898544573
314	V314A	0.323083736
314	V314G	0.87532591
314	V314D	0.953794332
314	V314I	0.532172719
314	V314L	0.613959384
315	T315S	1.206458283
315	T315A	1.28926502
315	T315I	0.442218528
315	T315T	0.5941292
315	T315P	0.06253291
316	I316K	0.006644068
316	I316V	2.012948534
316	I316M	0.183437796
316	I316T	0.026098855
316	I316L	0.595933273
316	I316I	0.313721179
317	G317R	0.030216232
317	G317G	0.797585767
317	G317E	0.026256077
318	E318E	0.628975246
318	E318G	0.028145531
318	E318V	0.013437703
318	E318K	0.610847785
319	C319R	0.028230187
319	C319C	0.162180778
319	C319G	0.015771425
319	C319Y	0.05666558
319	C319S	0.038083568
319	C319W	0
320	P320L	0.098689391
320	P320R	0.015161609
320	P320S	0.068271484
320	P320P	1.30088861
321	K321Q	0.180223751

321	K321K	0.234578892
321	K321I	0.577720278
321	K321R	1.07775951
321	K321N	0.160657036
321	K321T	0.201195839
321	K321_	0.036154604
321	K321E	0.06799865
322	Y322Y	1.513373906
322	Y322_	0.094766309
322	Y322C	0.395166767
322	Y322F	1.333762221
322	Y322H	0.106412928
322	Y322N	0.094623657
323	V323V	1.601994768
323	V323D	0.012981725
323	V323G	0
323	V323A	0.028017207
323	V323L	0.017568213
323	V323I	1.331916772
324	R324K	2.290209488
324	R324W	0.011928607
324	R324G	0.200608816
324	R324T	0.410190284
324	R324R	0.540082759
325	S325T	0.552657502
325	S325R	0.027157453
325	S325S	1.718406591
325	S325G	0.075721018
325	S325C	0.16901554
325	S325N	0.038938349
326	T326A	0.621708208
326	T326I	0.868624139
326	T326T	0.617229736
326	T326S	2.720517933
327	K327T	0.04364799
327	K327Q	0.023314281
327	K327R	0.33425761
327	K327_	0.024748533
327	K327E	0.119198617
327	K327I	0.012154561
327	K327K	0.55727306
327	K327N	0.208468002
328	L328S	0.024708507
328	L328W	0.029339748
328	L328_	0.007192259
328	L328L	1.486736274
328	L328M	0.044647443
329	R329T	0.109887271
329	R329K	1.148826565
329	R329G	0.143571308
329	R329W	0.009192488
329	R329S	0.017297692

329	R329R	5.151346287
330	M330T	0.099222101
330	M330V	0.083235739
330	M330K	0.01567732
330	M330I	0.098420401
330	M330L	0.180447746
331	V331V	0.987233119
331	V331D	0.005936108
331	V331L	0
331	V331I	0.09717806
331	V331A	0.242077751
332	T332T	1.124661644
332	T332P	0.013405712
332	T332S	0.085919931
332	T332R	0.009930157
332	T332I	0.090101289
332	T332A	0.112646952
333	G333R	0.048777856
333	G333E	0.192641512
333	G333G	0.373255379
333	G333A	0
334	L334L	0.813062585
334	L334V	0.720702996
334	L334Q	0.013649452
334	L334P	1.214571154
335	R335K	0.112060628
335	R335R	0.549248525
335	R335_	0.021122811
335	R335G	0.058021875
335	R335S	0.01255487
336	N336Y	0.003249869
336	N336S	0.036330087
336	N336I	0.002341609
336	N336D	0.031230321
336	N336N	1.892375176
336	N336K	0
337	I337F	4.827625925
337	I337N	0.058092391
337	I337M	6.474563666
337	I337L	4.479534681
337	I337I	0.640688709
337	I337V	1.462822988
337	I337T	4.587935946
338	P338A	0.128697377
338	P338S	0.066483992
338	P338P	1.13416702
338	P338L	0.184437249
339	S339T	0.211206446
339	S339S	0.540363908
339	S339P	1.224488302
339	S339F	0.12958125
339	S339C	0.015320813

340	I340T	0.085249772
340	I340V	20.2160828
340	I340I	0.402135621
340	I340N	0.026440609
340	I340F	0.131373634
341	Q341L	0.074562924
341	Q341R	0.534435093
341	Q341_	0.058582107
341	Q341H	0.016851548
341	Q341Q	1.502865936
342	Y342H	0.158353826
342	Y342F	0.374592193
342	Y342_	0.034470899
342	Y342Y	1.353668694
342	Y342C	0.114099675
342	Y342N	0.044321804
343	R343K	0.762558994
343	R343S	0.035172088
343	R343_	0.027370645
343	R343G	0.093161263
343	R343R	2.732944702
344	G344S	0.066265908
344	G344R	0
344	G344G	0.773626736
344	G344D	0.025378344
345	L345R	0.015096802
345	L345Q	0.024126857
345	L345P	0.206220717
345	L345L	1.594808199
346	F346Y	0.02543795
346	F346L	0.641756429
346	F346I	0.753457492
346	F346F	0.804666985
346	F346C	0.024749956
346	F346S	0.012593549
347	G347G	0.208351365
347	G347E	0.046015203
347	G347R	0.696062058
348	A348T	0.070000555
348	A348V	0.080280223
348	A348A	1.98498422
348	A348G	0.049957661
349	I349S	0.012639183
349	I349V	1.398220051
349	I349T	0.034518695
349	I349F	0.020028656
349	I349I	1.26664598
349	I349N	0.007969978
349	I349L	0.067957507
350	A350P	0.007058245
350	A350V	0.039051008
350	A350A	2.807073145

350	A350G	0.010573504
350	A350T	0.20446498
351	G351A	0.040499929
351	G351D	0.045549685
351	G351G	0.315479304
351	G351S	0.065508085
352	F352S	0.045216511
352	F352Y	0.025650288
352	F352F	0.35128447
352	F352I	0.014822489
352	F352V	0.020882776
352	F352L	0.038928956
353	I353I	1.600728594
353	I353M	0.079090642
353	I353N	0.012301865
353	I353F	0.105366587
353	I353T	0.053210483
353	I353V	1.905853188
354	E354A	0.021432778
354	E354D	0.037359832
354	E354E	0.333992169
354	E354G	0.064150607
354	E354K	0.063678011
354	E354V	0.031070095
355	G355E	0.063743803
355	G355G	0.446129661
355	G355A	0.02403069
355	G355R	0.011753932
356	G356E	0.061956368
356	G356G	0.301703244
356	G356A	0
356	G356R	0.02194952
357	W357S	0
357	W357R	0.020697772
357	W357_	0.044178268
357	W357C	0
357	W357G	0
358	T358T	0.231622209
358	T358P	3.349491272
358	T358A	0.061121483
358	T358I	0.419583274
358	T358S	0.493073484
359	G359A	0.214327776
359	G359E	0.168069856
359	G359G	0.210476043
359	G359R	0.093967558
360	M360L	0.341932143
360	M360K	0.009285316
360	M360I	0.034743608
360	M360V	0.03605196
360	M360T	0.015666521
361	I361T	0.072432816

361	I361V	1.541407177
361	I361L	0.366207163
361	I361I	0.768426784
361	I361K	1.045926478
361	I361M	0.534364863
362	D362D	1.958231436
362	D362A	0.035638352
362	D362V	0.013245323
362	D362N	0.027969393
362	D362E	0.212289746
362	D362G	0.036420944
363	G363R	0.124972345
363	G363E	0.0553831
363	G363G	0.332794464
364	W364G	0.025880232
364	W364_	0.039512273
364	W364S	0
364	W364R	0.004575212
364	W364C	0.008491951
365	Y365H	0.119180592
365	Y365N	0.02095223
365	Y365C	0.028629996
365	Y365D	0
365	Y365F	0.017835376
365	Y365Y	0.517668764
365	Y365_	0.017123297
366	G366G	1.065984269
366	G366D	0.048307144
366	G366S	0.051731464
366	G366R	0
367	Y367C	0.068310327
367	Y367F	0.439216637
367	Y367H	0.02235632
367	Y367N	0.005389103
367	Y367Y	0.063546046
367	Y367_	0.011863568
368	H368L	0.306603761
368	H368R	0.241173058
368	H368Q	0.42271662
368	H368Y	0.456288033
368	H368D	0
368	H368H	0.9525596
369	H369P	0.277641928
369	H369Y	0.376549995
369	H369D	0.049407715
369	H369L	0.057947618
369	H369Q	1.134612782
369	H369R	0.013034431
369	H369H	0.82863153
370	Q370_	0.041765551
370	Q370L	0.064265325
370	Q370E	4.643485162

370	Q370R	0.400760488
370	Q370P	0
370	Q370Q	0.685200728
370	Q370H	0.212034297
371	N371Y	0.008586736
371	N371S	0.028048585
371	N371T	0.059399895
371	N371K	0.018860589
371	N371I	0.014891112
371	N371N	0.867341485
371	N371D	0.069052696
372	E372V	0.030745619
372	E372D	1.645976083
372	E372E	0.645893299
372	E372G	1.060557626
372	E372K	0.055687402
373	Q373P	0.056500067
373	Q373E	0.266701098
373	Q373H	0.011322601
373	Q373R	0.106340647
373	Q373Q	0.419577861
373	Q373_	0.073530053
373	Q373L	0.248058629
374	G374R	0.045857587
374	G374E	0.061406863
374	G374G	0.679502839
375	S375L	0.079920239
375	S375T	1.424599612
375	S375S	0.315422347
375	S375P	0.014139683
376	G376G	1.18145787
376	G376D	0.051843539
376	G376A	2.61165848
376	G376R	0
376	G376S	0.253751494
377	Y377C	0.014099198
377	Y377F	0.016223429
377	Y377D	0
377	Y377Y	0.691708781
377	Y377_	0.027686106
377	Y377H	0.051377032
377	Y377N	0.004396374
378	A378A	1.04492189
378	A378G	0.00670969
378	A378V	0.692581188
378	A378T	0.090893413
379	A379A	0.186062868
379	A379G	0.038718371
379	A379T	0.020053837
379	A379V	0.136546487
380	D380H	0.054348487
380	D380V	0.014597282

380	D380A	0
380	D380D	2.158598682
380	D380E	0.018727641
380	D380N	0.07579639
380	D380G	0.048306732
381	Q381L	0.613641997
381	Q381_	0.030872196
381	Q381H	0.394150002
381	Q381R	1.748570574
381	Q381P	0
381	Q381Q	1.308092975
382	K382N	0.009769416
382	K382I	0.151432343
382	K382K	0.389617094
382	K382E	0.406079009
382	K382_	0.019790766
382	K382Q	0.190928235
382	K382R	0.328992747
382	K382T	0.06172494
383	S383G	0.142921893
383	S383C	0.353196346
383	S383N	0.112452125
383	S383T	2.598985382
383	S383R	0.048979642
383	S383S	1.000489428
384	T384A	0.022461109
384	T384I	0.021750095
384	T384T	0.666847433
384	T384P	0.007246465
384	T384R	0.019092823
384	T384S	0.05383975
385	Q385E	0.044201458
385	Q385R	0.043679684
385	Q385P	0.041806528
385	Q385Q	0.354950843
385	Q385_	0.024161321
385	Q385H	0.015050649
385	Q385L	0.015738422
386	N386H	0.188632952
386	N386I	1.023456608
386	N386K	9.758055688
386	N386N	1.07503366
386	N386D	0.578843771
386	N386Y	0.616253954
386	N386S	1.369274526
386	N386T	0.745021324
387	A387G	0
387	A387V	0.105170774
387	A387A	0.360270085
387	A387P	0.009881543
387	A387T	0.011657351
388	I388N	0.010447015

388	I388T	0.019845834
388	I388L	0.165236412
388	I388I	8.564714665
388	I388F	0.164720859
388	I388V	1.55011172
388	I388S	0.046999563
389	N389T	0.088793435
389	N389Y	0.192005037
389	N389D	0.577851988
389	N389N	0.420838908
389	N389I	0.099398161
389	N389K	0.067965655
389	N389S	0.380527367
390	G390R	7.376041968
390	G390G	0.607349518
390	G390E	0.066434627
390	G390A	0.663715251
391	I391V	1.106851599
391	I391T	0.034935944
391	I391F	0.002388593
391	I391I	0.4999246
391	I391N	0
391	I391L	0.488501681
392	T392I	0.022702744
392	T392A	0.122761436
392	T392T	0.757604155
392	T392S	0.880841495
392	T392R	0.229913148
392	T392P	0.010254431
393	N393I	0.410506341
393	N393N	1.726904514
393	N393D	0.038812843
393	N393Y	0.021727132
393	N393S	0.068271361
393	N393T	0.420219969
393	N393K	0.022157963
393	N393H	0.153458005
394	K394E	0.036571777
394	K394M	0.012032875
394	K394K	0.571791804
394	K394_	0.009810996
394	K394T	0.06641199
394	K394R	0.063237337
394	K394Q	0.012277805
395	V395M	0.17745574
395	V395E	0.0140291
395	V395A	0.016370463
395	V395L	0.007805757
395	V395V	2.763097103
396	N396T	0.059528883
396	N396D	0.01965266
396	N396I	0.00432025

396	N396H	0.016838133
396	N396K	0.007653697
396	N396Y	0.005806253
396	N396S	0.108282276
396	N396N	2.244818644
397	S397F	0.079277333
397	S397P	0.012895627
397	S397T	1.125642552
397	S397S	1.487842715
398	V398V	0.70849409
398	V398G	0.062047462
398	V398D	0.022080889
398	V398I	0.231957444
398	V398A	0.019296091
399	I399I	0.970673833
399	I399N	0.022669127
399	I399F	0.005102465
399	I399V	0.438844947
399	I399T	0.041518715
400	E400E	0.208789472
400	E400V	0.019122172
400	E400Q	0.245060353
400	E400K	0.148231286
400	E400D	2.570489688
400	E400G	0.415359417
401	K401I	0.21212019
401	K401R	1.018581797
401	K401_	0.00851403
401	K401E	0.05018814
401	K401K	1.161650452
401	K401N	0.058801907
402	M402V	0.063823186
402	M402T	0.0523339
402	M402L	1.132203719
402	M402K	0.194924137
402	M402I	0.270942597
403	N403D	0.100190758
403	N403K	0.038644895
403	N403I	0.028817995
403	N403N	0.270462591
403	N403S	0.233052482
403	N403T	0.190829598
403	N403Y	0.171302783
404	T404A	2.596416003
404	T404I	0.208950372
404	T404P	0.044697175
404	T404S	3.699645743
404	T404T	0.553031565
405	Q405H	0.616901364
405	Q405L	0.01887916
405	Q405_	0.02602359
405	Q405R	0.335007993

405	Q405Q	0.990898202
406	F406V	0.667055414
406	F406S	0.092339243
406	F406Y	0.685970108
406	F406F	0.852855299
406	F406L	0.614545351
406	F406I	0.530644933
407	T407R	1.400812859
407	T407A	0.819480822
407	T407S	1.128814339
407	T407T	0.979414282
407	T407I	0.533733202
407	T407P	0.645293601
408	A408T	0.196722629
408	A408P	0.156421502
408	A408V	0.156183804
408	A408A	0.752918518
408	A408G	0.313924694
409	V409M	0.994358285
409	V409L	0.175308941
409	V409A	0.107361763
409	V409G	0.113020001
409	V409V	0.237240461
409	V409E	0.147898808
410	G410S	0.157468353
410	G410R	0.121816389
410	G410G	1.113120159
410	G410D	0.322043227
410	G410A	0.827342537
411	K411N	0.478194965
411	K411T	0.094971404
411	K411R	0.018898273
411	K411_	0.019817407
411	K411E	0.017761247
411	K411I	0.08187659
411	K411K	0.257677575
412	E412V	0.93594955
412	E412A	0.099514466
412	E412G	0.441152723
412	E412E	0.262931886
412	E412D	0.534305956
412	E412K	0.221357885
413	F413I	0.008763341
413	F413L	0.025076176
413	F413C	0.017858626
413	F413F	0.311782683
413	F413Y	0.664689158
413	F413S	0.023117832
414	N414T	1.218889387
414	N414Y	0.004449634
414	N414S	0.498191845
414	N414K	0.124666946

414	N414H	0.034699123
414	N414I	0.055031735
414	N414N	0.347955826
414	N414D	0.0998252
415	N415K	0.016252371
415	N415D	0.298741371
415	N415Y	1.235824872
415	N415S	1.242475768
415	N415T	0.242324071
415	N415I	0.267709059
415	N415H	0.209381125
415	N415N	2.909900508
416	L416V	0.014374016
416	L416_	2.139412128
416	L416S	0.046386565
416	L416I	0.277774838
416	L416L	1.32918547
416	L416F	0.195758533
417	E417D	0.006892803
417	E417E	3.309812062
417	E417G	0.171722291
417	E417K	1.361720022
417	E417V	0.020976844
418	K418R	1.667285981
418	K418Q	0.064320326
418	K418T	0.097726689
418	K418_	0.01182251
418	K418E	0.035635309
418	K418K	0.332544214
418	K418I	0.04295817
418	K418N	0.016778183
419	R419R	0.240742449
419	R419T	0
419	R419W	0.003006809
419	R419G	0.256132061
419	R419K	0.280843347
420	M420T	0.008016392
420	M420R	0.033029011
420	M420K	0.069507966
420	M420V	0.427968243
420	M420L	0.095987003
420	M420I	0.572666987
421	E421K	0.557649159
421	E421E	0.981386693
421	E421D	0.718899993
421	E421G	0.336224157
421	E421A	0.127987419
421	E421V	0.003022229
422	N422Y	0.211571672
422	N422S	2.301546469
422	N422K	0.168560427
422	N422I	0.156725334

422	N422N	1.370123513
422	N422D	0.201978485
423	L423_	0.011334197
423	L423S	0.089968044
423	L423I	0.008191195
423	L423L	0.944744545
423	L423F	0
424	N424D	0.080690847
424	N424I	0.010160942
424	N424K	0
424	N424N	0.760647309
424	N424S	0.058571398
424	N424Y	0.242836919
425	K425E	0.207749723
425	K425N	0.071323414
425	K425K	2.260962001
425	K425R	0.480967133
425	K425_	0.031277071
425	K425I	0.034229074
426	K426T	0.016838133
426	K426R	0.162510001
426	K426Q	0.078720399
426	K426_	0.004533343
426	K426E	0.034943697
426	K426N	0.01010288
426	K426K	0.75137633
426	K426I	0.007963982
427	V427V	0.994881213
427	V427D	0.032687792
427	V427I	1.273388047
427	V427A	0.026645617
428	D428G	0.048620771
428	D428E	0.190937824
428	D428D	0.561008792
428	D428N	0.476675306
428	D428V	0.044623761
429	D429D	0.601344826
429	D429E	0.057082053
429	D429G	0.118055295
429	D429N	0.209728755
429	D429V	0.003324349
430	G430E	0.210072126
430	G430G	0.242374035
430	G430A	0.071050602
430	G430R	0.115699126
431	F431S	0.019281681
431	F431Y	0.013105574
431	F431C	0.075234211
431	F431F	0.348433578
431	F431I	0.072985293
431	F431L	0.083306931
432	L432P	0.060825757

432	L432Q	0.08982936
432	L432R	1.03487835
432	L432L	1.009085365
433	D433A	0.017858626
433	D433G	0.247167527
433	D433E	0.40908255
433	D433D	0.239922002
433	D433V	0.027541863
433	D433N	0.524553155
434	I434N	0.022907328
434	I434I	1.494872439
434	I434F	0.015189037
434	I434V	1.025469
434	I434T	0.061535183
434	I434S	0.101846349
435	W435_	0.023437694
435	W435R	0.02666599
435	W435S	0
435	W435C	0
436	T436A	0.12504103
436	T436P	0
436	T436R	0.018961099
436	T436I	0.050072816
436	T436S	0.0379435
436	T436T	0.417386649
437	Y437Y	2.263061252
437	Y437_	0.005617512
437	Y437C	0.091002944
437	Y437F	0.350100258
437	Y437N	0.0140291
437	Y437H	0.102423956
438	N438N	0.900272243
438	N438H	0.013393969
438	N438K	0.002983973
438	N438D	0.102157007
438	N438Y	0.02889947
438	N438T	0.128799011
438	N438I	0.011119522
438	N438S	0.183004146
439	A439T	0.104804422
439	A439V	0.330973599
439	A439P	0.011119522
439	A439A	0.666557783
439	A439G	0.007100418
440	E440V	0.003513794
440	E440K	0.035620778
440	E440E	0.100517051
440	E440G	0.088096568
440	E440A	0.013455261
440	E440D	0.067040099
441	L441_	0.011176143
441	L441F	1.457842829

441	L441L	0.988287968
441	L441M	0.096524331
441	L441S	0.103273615
441	L441W	0.013134898
442	L442V	0.06038706
442	L442S	0.016015582
442	L442L	0.217035859
442	L442I	0.822118647
442	L442_	0.015501113
442	L442F	0.170738483
443	V443V	0.75687583
443	V443G	0
443	V443D	0.01420872
443	V443A	0.046416249
443	V443L	0.053374397
443	V443I	0.539194084
444	L444Q	0.040864668
444	L444L	0.487665575
444	L444V	0.03374571
444	L444P	0.055388205
445	L445R	0.04025804
445	L445L	1.313977576
445	L445V	0.029893156
445	L445P	0.038967337
445	L445Q	0.012876257
446	E446K	0.113126213
446	E446G	0.071590971
446	E446D	0.03077993
446	E446E	1.055446596
446	E446V	0.008283269
447	N447H	0.052406515
447	N447N	0.650942271
447	N447Y	0.008530913
447	N447D	0.048340428
447	N447K	0.015360493
447	N447I	0.028940329
447	N447S	0.027694755
448	E448D	0.094849081
448	E448E	0.381159004
448	E448G	0.055623891
448	E448K	0.073042516
448	E448V	0.056576677
449	R449K	0.197020786
449	R449G	0.105322093
449	R449R	1.176676314
449	R449W	0.03745423
450	T450A	0.033609264
450	T450I	0.017732495
450	T450T	0.231558969
450	T450S	0.046358463
451	L451_	0.017621375
451	L451W	0.07811883

451	L451V	0.023360331
451	L451S	0.01257377
451	L451M	0.004982463
451	L451L	0.489317434
452	D452V	0.020645691
452	D452N	0.063535133
452	D452G	0.041595012
452	D452D	0.154964152
452	D452E	0.031347544
453	F453S	0.070263972
453	F453Y	0.104542168
453	F453F	1.334997737
453	F453L	0.869526864
453	F453I	0.060883355
454	H454Q	0.026055242
454	H454H	2.437072863
454	H454L	0.00494029
454	H454R	0.0346188
454	H454Y	0.023691427
455	D455A	0.389741061
455	D455G	0.082460567
455	D455E	0.05516657
455	D455D	0.85251921
455	D455H	0
455	D455N	0.080175704
455	D455V	0.015050973
456	L456S	0.043987907
456	L456L	0.843407035
456	L456I	0.577736225
456	L456F	0.228658183
456	L456_	0.020220654
457	N457K	0.005600667
457	N457I	0.007662024
457	N457H	0.096078779
457	N457N	0.204607155
457	N457D	0.018047045
457	N457Y	0.014092508
457	N457S	0.458101101
457	N457T	0.061296189
458	V458G	0
458	V458L	0.168938902
458	V458M	0.037326378
458	V458A	0.010584448
458	V458E	0.005293767
458	V458V	2.159916262
459	K459_	0.007244097
459	K459Q	0.466633972
459	K459R	0.243525045
459	K459T	0.055811254
459	K459E	0.03889889
459	K459K	0.682174896
459	K459M	0.008732316

460	N460Y	0.016159917
460	N460N	1.079949899
460	N460S	0.248465474
460	N460D	0.765409531
460	N460I	0.016481498
460	N460K	0.182308678
461	L461Q	0.017355939
461	L461L	1.276594055
461	L461V	1.7965821
461	L461P	0.021568472
461	L461R	0.011992733
462	Y462_	0.007227062
462	Y462Y	0.491602513
462	Y462S	0.012156907
462	Y462N	0.016426151
462	Y462H	0.039315372
462	Y462D	0
462	Y462F	0.24286676
462	Y462C	0.072198801
463	E463K	0.065203158
463	E463V	0.044923917
463	E463Q	0.098537594
463	E463G	0.197430228
463	E463E	0.240289828
463	E463A	4.377247762
464	K464_	0.013635774
464	K464K	0.773993902
464	K464I	0.013766215
464	K464N	0.447253872
464	K464E	0.201893044
464	K464R	0.263233512
464	K464T	0.418798075
465	V465V	0.627353529
465	V465L	0.022066628
465	V465I	0.404988649
465	V465G	0.050841978
465	V465E	0.04248749
465	V465A	0.301431349
466	K466E	0.059356015
466	K466K	2.109549316
466	K466I	0.003118788
466	K466N	0.336663927
466	K466R	0.514208554
466	K466Q	0.659838957
466	K466T	0.029057972
466	K466_	0.010668279
467	S467T	0.012410176
467	S467N	0.296373993
467	S467C	0.240937749
467	S467G	0.518343122
467	S467S	0.613829619
467	S467R	0.098031569

468	Q468Q	0.941477804
468	Q468R	0.381304918
468	Q468_	0.012017116
468	Q468L	0.959953255
468	Q468H	1.370110434
469	L469L	0.762886318
469	L469I	1.235900245
469	L469V	0.0779577
469	L469S	0.039654316
469	L469F	0.010574692
469	L469_	0.001889266
470	K470_	0.006297288
470	K470T	0.178139569
470	K470R	0.172805629
470	K470M	0.1335967
470	K470K	1.215970386
470	K470E	0.8037287
471	N471D	0.099736039
471	N471N	0.903675162
471	N471I	0.100087499
471	N471H	0
471	N471T	0.326284718
471	N471S	1.471849405
471	N471Y	0.310122955
471	N471K	0.093280824
472	N472S	0.044892744
472	N472T	0.048676386
472	N472K	0.011139361
472	N472I	0.007626715
472	N472N	0.108184553
472	N472D	0.193942444
472	N472Y	0.011680165
473	A473V	0.32305012
473	A473A	0.79988957
473	A473G	0.066820885
473	A473T	0.040299629
474	K474E	0.61998191
474	K474I	0.558573692
474	K474K	1.947555791
474	K474N	0.234117868
474	K474Q	1.506816618
474	K474R	0.923843538
474	K474T	0.032278566
474	K474_	0.006142888
475	E475V	0.006895821
475	E475K	0.137758365
475	E475E	0.322997536
475	E475G	0.198017219
475	E475D	0.266346777
476	I476L	0.175896234
476	I476I	0.600816009
476	I476F	0.118709606

476	I476T	0.422038502
476	I476V	0.534801694
476	I476N	0.43731488
477	G477G	1.140884343
477	G477R	0.084782453
477	G477E	0.239231253
478	N478T	0
478	N478S	0.058141933
478	N478D	0.074440308
478	N478Y	0.033826199
478	N478H	0.225134726
478	N478I	0.012298828
478	N478K	0.248459214
478	N478N	3.277969623
479	G479A	0.052819472
479	G479G	0.390026039
479	G479E	0.04107078
479	G479R	0.32338704
480	C480W	0.113267989
480	C480C	0.297545599
480	C480R	0.031267
480	C480Y	0.050003153
480	C480_	0.008384055
480	C480G	0.044693399
480	C480S	0.005076788
481	F481L	0.244124228
481	F481Y	0.611929099
481	F481S	0.020667767
481	F481V	0.137758365
481	F481C	0.024985586
481	F481I	0.03447046
481	F481F	0.896334958
482	E482V	0.548384865
482	E482K	0.246153706
482	E482G	0.214720038
482	E482D	0.313446423
482	E482E	0.551925246
483	F483I	0.009146825
483	F483L	0.045418995
483	F483S	0.023929857
483	F483V	0.111800565
483	F483Y	0.226568148
483	F483F	0.741763376
484	Y484C	0.03982787
484	Y484_	0.01329315
484	Y484Y	0.073036134
484	Y484F	1.156299536
484	Y484N	0.106210523
484	Y484H	0.74850886
485	H485P	0.037341095
485	H485L	0.020754052
485	H485H	0.801449648

485	H485R	0.019050948
485	H485D	0.009511478
485	H485Q	0
485	H485Y	0.006066538
486	K486M	0.229269344
486	K486N	0.100549791
486	K486K	1.425488031
486	K486E	0.757448869
486	K486_	0.003566855
486	K486T	0.590590558
486	K486Q	1.145206683
486	K486R	0.644286897
487	C487C	0.482368759
487	C487Y	0.040442749
487	C487_	0.011361996
487	C487R	0.011318458
487	C487W	0
487	C487S	0.00969237
488	D488V	0.097709926
488	D488N	0.448446933
488	D488H	2.042692959
488	D488E	0.066429649
488	D488D	0.294912001
488	D488G	0.336696721
488	D488A	0.099644496
489	N489Y	0.013097116
489	N489H	0.024145831
489	N489K	0.072537877
489	N489N	0.868506283
489	N489D	0.955943526
489	N489S	0.093839589
489	N489I	0.002192199
490	E490E	0.52019755
490	E490D	0.578250085
490	E490G	0.557239444
490	E490V	0.532814709
490	E490K	1.74165649
491	C491S	0.004806809
491	C491Y	0.061635219
491	C491G	0.051353434
491	C491W	0
491	C491R	0.048564962
491	C491C	0.974038426
492	M492R	0.071965463
492	M492V	0.313170017
492	M492T	0.046008146
492	M492K	0.003932844
492	M492I	2.145432037
492	M492L	0.055894146
493	E493Q	0.86058692
493	E493D	1.247367203
493	E493E	0.592454119

493	E493G	0.47749001
493	E493V	0.444920776
493	E493K	0.133715771
494	S494R	0.559036667
494	S494N	0.283527704
494	S494G	0.702935282
494	S494C	0.625504651
494	S494T	1.887402142
494	S494S	1.138657104
495	V495L	0.045418211
495	V495I	0.565928349
495	V495A	0.111451794
495	V495E	0.016630196
495	V495V	0.887752967
496	R496G	0.028190902
496	R496K	2.91482646
496	R496R	0.362784819
496	R496S	0.014737421
496	R496_	0.008124184
497	N497Y	2.342918939
497	N497T	1.247732817
497	N497S	0.623256297
497	N497N	1.000309319
497	N497K	1.205342432
497	N497I	4.141824041
497	N497D	3.996408362
498	G498G	0.304799707
498	G498E	0.157991297
498	G498A	0.027322914
498	G498R	0.18788754
499	T499I	6.550408268
499	T499A	0.782130863
499	T499S	1.129258885
499	T499T	0.670570669
500	Y500N	0.01325761
500	Y500H	0.072760975
500	Y500F	0.003047489
500	Y500C	0.039212782
500	Y500_	0.025084653
500	Y500Y	1.313287623
500	Y500S	0.044122417
501	D501V	4.992948444
501	D501D	0.443040535
501	D501E	0.841608023
501	D501G	0.185916364
501	D501N	0.388846386
502	Y502C	0.126583322
502	Y502Y	1.676167369
502	Y502_	0.011270027
502	Y502F	1.4091113
502	Y502N	0.096028773
502	Y502H	0.528070508

503	P503L	0.652452961
503	P503S	0.767415709
503	P503R	0.333378485
503	P503P	0.809884776
504	K504T	0.127276202
504	K504Q	0.427302559
504	K504R	0.657992602
504	K504E	0.147395658
504	K504N	0.418655491
504	K504I	0.298856978
504	K504K	0.811105188
504	K504_	0.006238217
505	Y505Y	0.528410527
505	Y505F	0.055842662
505	Y505C	0.098360833
505	Y505N	0.020364192
505	Y505H	0.290826115
505	Y505_	0.006954933
506	S506_	0.016746302
506	S506S	1.126341156
506	S506P	0.024242377
506	S506T	0.702211929
506	S506L	0.580417742
507	E507A	0.16937624
507	E507G	0.233860509
507	E507D	5.502150529
507	E507E	0.556319506
507	E507K	0.456044922
507	E507V	0.120120109
508	E508D	0.069248975
508	E508A	0
508	E508G	0.042595722
508	E508E	1.155029049
508	E508K	0.495599569
508	E508V	0.026694885
509	S509S	0.249798142
509	S509L	1.384486659
509	S509P	0.038814648
509	S509T	0.166365618
510	K510E	0.642939119
510	K510K	1.360989912
510	K510M	0.493292084
510	K510N	0
510	K510Q	0.251220051
510	K510R	1.927750284
510	K510T	0.031946791
510	K510_	0.011588733
511	L511L	0.685324758
511	L511M	0.152660212
511	L511S	0.318182824
511	L511V	0.211424907
511	L511W	0.011818504

511	L511_	0.009097405
512	N512S	1.540622162
512	N512D	0.227215084
512	N512N	1.280134426
512	N512I	1.159278327
512	N512T	0.030783081
512	N512Y	0.114861876
513	R513W	0.094361962
513	R513G	0.232997001
513	R513K	1.416618114
513	R513T	0
513	R513R	0.38824628
514	E514V	0.034307363
514	E514K	0.523977534
514	E514G	0.361238291
514	E514D	0.240429035
514	E514E	0.109796947
515	K515R	0.575348897
515	K515K	0.130587344
515	K515Q	0.048112661
515	K515T	0.498672951
515	K515_	0.008853872
515	K515E	7.822937115
515	K515M	0.134903294
516	I516T	0.029504245
516	I516V	0.158143444
516	I516I	0.415357715
516	I516K	0.008929687
516	I516L	0.139875737
516	I516M	0.101585108
517	D517G	0.684646206
517	D517N	0.274750256
517	D517D	1.083840775
517	D517E	0.95325902
517	D517A	0.100975013
517	D517V	2.713823162
518	G518G	0.683503214
518	G518A	0
518	G518R	5.314527136
518	G518E	0.301304939
519	V519V	2.141209032
519	V519L	0.021962217
519	V519M	0.130448498
519	V519A	0.201124152
519	V519E	0.013500536
520	K520I	0.122835685
520	K520N	0.129300426
520	K520E	0.13078373
520	K520R	0.687826038
520	K520K	0.934868826
520	K520_	0.020758425
521	L521S	0.076657391

521	L521_	0.009394334
521	L521M	0.37835604
521	L521L	1.121402887
522	E522V	0.074240573
522	E522G	0.218868051
522	E522E	0.656938632
522	E522D	0.397570085
522	E522K	0.095553561
523	S523S	0.315292036
523	S523T	1.312909024
523	S523P	0.858261832
523	S523_	0.008031675
523	S523A	0.04100162
523	S523L	0.095037803
524	M524L	1.066145444
524	M524I	1.066474977
524	M524K	0.111764697
524	M524T	0.167060168
524	M524V	0.15983517
524	M524R	0.060993344
525	G525E	1.124799413
525	G525G	2.493101672
525	G525R	0.022556266
526	V526V	0.517238389
526	V526L	2.627571413
526	V526M	2.890320812
526	V526E	0.157805312
526	V526G	0.055832725
526	V526A	0.868292226
527	Y527H	0.816625499
527	Y527N	0.24052495
527	Y527C	0.251172236
527	Y527F	1.025180152
527	Y527Y	0.224272734
527	Y527_	0.021486383
528	Q528L	0.061512651
528	Q528_	0.019858734
528	Q528Q	0.725984518
528	Q528R	0.129162111
529	I529V	0.400735454
529	I529T	0.051946247
529	I529F	0.303452582
529	I529N	0.03514649
529	I529I	0.93086493
530	L530Q	0.011359831
530	L530P	0.028741059
530	L530R	0.03937267
530	L530L	0.0973013
531	A531T	0.36927878
531	A531A	0.201921491
531	A531G	0.054268464
531	A531V	0.099236822

532	I532N	0.008083326
532	I532M	0.008590401
532	I532I	0.669905728
532	I532F	0.281405067
532	I532V	0.251705521
532	I532T	0.110343297
533	Y533F	0.345895303
533	Y533C	0.087203338
533	Y533N	0.044319636
533	Y533D	0.007717938
533	Y533_	0.019392317
533	Y533Y	2.653538726
533	Y533S	0.05933825
533	Y533H	0.196009069
534	S534T	0.038937716
534	S534S	1.576582832
534	S534P	0.064188441
534	S534A	6.540048783
534	S534L	0.011611723
535	T535A	0.309241195
535	T535I	0.037035818
535	T535S	2.237670293
535	T535P	0.057439646
535	T535T	1.160640182
536	V536V	0.160750365
536	V536I	0.299886378
536	V536D	0.005015729
536	V536G	0
536	V536A	0.886642022
537	A537A	2.091320523
537	A537G	0.064431755
537	A537P	0.010805538
537	A537T	0.296487326
537	A537V	0.065641032
538	S538R	0.081151881
538	S538S	0.523044903
538	S538T	0.176863917
538	S538G	0.127793543
538	S538N	0.122561514
538	S538C	0.614504206
539	S539P	0.024443328
539	S539S	0.880620604
539	S539T	0.093387566
539	S539L	0.021277572
540	L540L	0.591979102
540	L540P	0.030251574
540	L540Q	0.001767071
540	L540V	0.781316332
540	L540R	0.052867081
541	V541A	0.16316428
541	V541G	0
541	V541E	0.010828403

541	V541M	0.51881897
541	V541V	0.295933173
542	L542P	0.059916704
542	L542R	0.045991688
542	L542F	1.72792305
542	L542H	0.017208381
542	L542L	0.649216465
543	L543W	0.366721639
543	L543S	0.769864099
543	L543_	0.019967636
543	L543L	1.07007686
543	L543M	0.395092516
544	V544I	0.270048861
544	V544V	0.010619251
544	V544A	0.072999626
544	V544D	0
544	V544G	0.308742105
545	S545A	0.066627818
545	S545F	0.018214799
545	S545S	0.649130451
545	S545P	0.116176029
545	S545T	0.035700466
546	L546L	0.164858741
546	L546V	0
546	L546P	0.141981107
546	L546R	0.178181643
546	L546Q	0.030186039
547	G547E	0.041031167
547	G547G	0.015598858
547	G547R	0.041690455
547	G547A	0.165149508
548	A548A	0.603898307
548	A548P	0
548	A548V	0.080613769
548	A548T	0.192121429
549	I549V	0.71761933
549	I549T	0.106891561
549	I549S	0.060641652
549	I549M	1.303330879
549	I549N	0.016677211
549	I549I	0.728003575
549	I549F	0.072999626
550	S550N	0.102081627
550	S550R	0.009716548
550	S550C	0.981189616
550	S550S	0.372416985
550	S550G	3.236838482
551	F551V	0.135108036
551	F551Y	0.418049476
551	F551F	1.720925408
551	F551S	0.077979583
551	F551L	0.080053879

551	F551I	0.595445327
552	W552R	0.035257911
552	W552S	0.008868696
552	W552G	0.25825508
552	W552_	0.06186753
553	M553L	0.444655515
553	M553V	0.144630484
553	M553K	0.035222396
553	M553I	0.055701221
553	M553R	0.110714891
553	M553T	0.046394709
554	C554S	0.203669369
554	C554C	0.685966464
554	C554G	0.120075919
554	C554R	0.020736549
554	C554W	0.129476737
554	C554Y	0.059342039
554	C554_	0.007413961
555	S555T	0.568370254
555	S555S	0.381797647
555	S555P	0.043374995
555	S555F	0.620191823
555	S555C	0
555	S555A	0
556	N556N	0.804786787
556	N556K	1.276836423
556	N556I	0.702448565
556	N556D	0.199407583
556	N556Y	0.031857753
556	N556S	0.212813101
557	G557R	0.274997497
557	G557E	0.116191365
557	G557G	0.778990751
557	G557A	0
558	S558F	0.070671896
558	S558A	0.12205154
558	S558T	0.551845488
558	S558P	0.060536844
558	S558S	2.86109834
559	L559L	0.292326063
559	L559M	0.609096959
559	L559V	0.082948395
559	L559_	0.00098956
559	L559S	0.034447913
560	Q560L	0.004930499
560	Q560R	0.22441459
560	Q560_	0.111201873
560	Q560E	0.060911208
